# Supplementary material for: The Costs of Close Contacts: Visualizing the Energy Landscape of Cell Contacts at the Nanoscale
Source: Biophys J. 2020 Jan 28;118(6):1261–9. doi: 10.1016/j.bpj.2020.01.019 (PMC7091464; doi:10.1016/j.bpj.2020.01.019)
Supplement: Document S1. Figs. S1–S15 and Table S1 [file mmc1.pdf]

Biophysical Journal, Volume *118*

Supplemental Information

The Costs of Close Contacts: Visualizing the Energy Landscape of Cell Contacts at the Nanoscale

Klara Kulenkampff, Anna H. Lippert, James McColl, Ana Mafalda Santos, Aleks Ponjavic, Edward Jenkins, Jane Humphrey, Alexander Winkel, Kristian Franze, Steven F. Lee, Simon J. Davis, and David Klenerman

Supporting Material

The costs of close contacts: Visualizing the energy landscape of cell contacts at the nanoscale

K. Kulenkampff [1]*, A. H. Lippert[1]*, J. McColl[1], M. A. Santos[2], A. Ponjavic[1], Edward Jenkins[2], Jane Humphrey[1], Alexander Winkel[3], Kristian Franze[3], S. F. Lee[1], S. J. Davis[2], D. Klenerman[1]

[1] Department of Chemistry, University of Cambridge, Cambridge, United Kingdom
[2] Radcliffe Department of Medicine and MRC Human Immunology Unit, John Radcliffe Hospital, University of Oxford, Oxford, United Kingdom
[3] Department of Physiology, Development and Neuroscience, University of Cambridge, Cambridge, United Kingdom

Suppl. Video 1: Measuring unspecific binding of QDots to cells in HILO mode

Suppl. Video 2A and 2B: QDots 605 diffusing in a bilayer and through a CD2rCD45 cell contact. (A) Raw video of QDots diffusing in bilayer. (B) Time-lapse of tracks colour-coded according to track speed (analysed with TrackMate by Fiji)

Suppl. Video 3: QDots 525 diffusing in a bilayer and through a CD2rCD45 cell contact

Suppl. Video 4A and 4B: Simulation of QDots diffusing freely in a bilayer and through a CD45 cell contact. (A) Tracks starting inside the cell contact. (B) Tracks starting outside the cell contact

Suppl. Table 1: Number of tracks attempting to enter or exit the cell contact and success to enter or exit.

Suppl. Fig. 1: Variation of bilayer intensity for different conditions

Suppl. Fig. 2: FCS measurements of protein densities

Suppl. Fig. 3: Unspecific binding of QDots to cells is minor.

Suppl. Fig. 4: Average tracklength in frames for each cell measured

Suppl. Fig. 5: Average signal-to-noise-ratio for each cell measured

Suppl. Fig. 6: QDots diffuse freely in bilayer (MSD plot)

Suppl. Fig. 7: Contacts are mediated by rat CD2 and rat CD48 constructs.

Suppl. Fig. 8: Shift of JD distribution between attempts, exit and enter

Suppl. Fig. 9: Energy penalties for eroded and dilated contact borders

Suppl. Fig. 10: Maps of number of counts and jump distance per pixel

Suppl. Fig. 11: ECDFs of overall jump distances in track image inside the contacts

Suppl. Fig. 12: Contact sizes between gap conditions similar

Suppl. Fig. 13: Negative correlation between success rate and spacer pixel intensity

Suppl. Fig. 14: JD distributions separated out via spacer intensity zones

Suppl. Fig. 15: Additional statistics for linear fit of diffusion constants in areas of various protein density

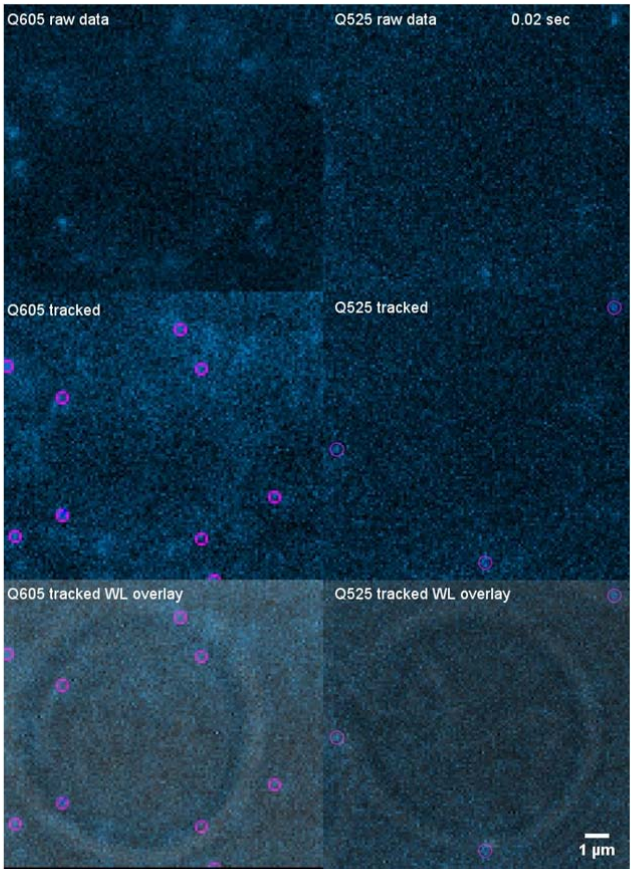

Supplementary Video 1: Measuring unspecific binding of QDots to cells in HILO mode. Detected QDots are marked with a purple circle.

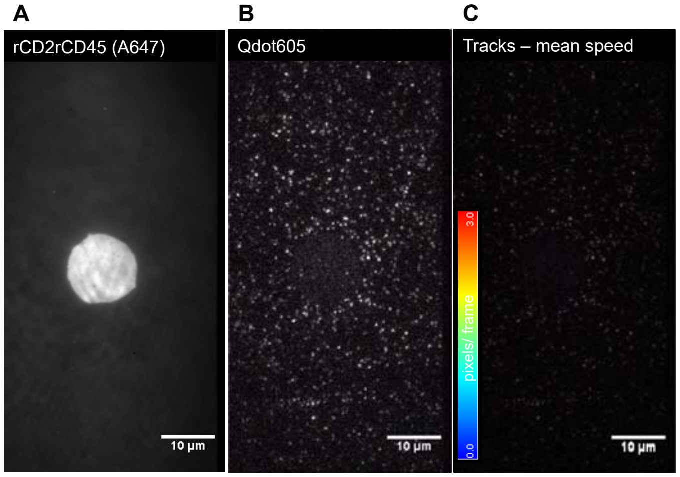

Supplementary Video 2: Raw data and tracks of Q605s attached to a SLB. Cell is immobilised by the protein spacer rCD2rCD45. (A) Average image over 500 frames of the labelled protein spacer. (B) Raw time-lapse data of the channel showing the QDots. (C) Time-lapse of tracks colour-coded according to track speed (analysed with TrackMate by Fiji).

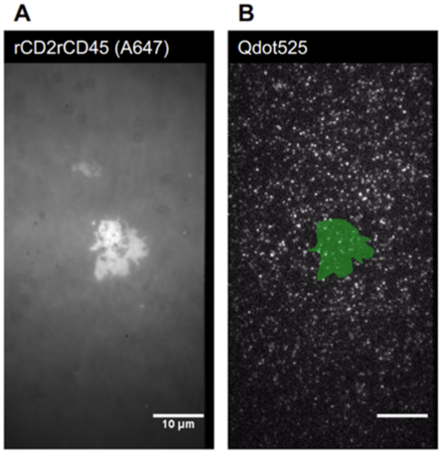

Supplementary Video 3: Raw data of Q525s attached to a SLB. Cell is again immobilised by the protein spacer rCD2rCD45. (A) Average image over 500 frames of the labelled protein spacer. (B) Raw time-lapse data of the channel showing the QDots. The green mask indicates the approximate position of the cell.

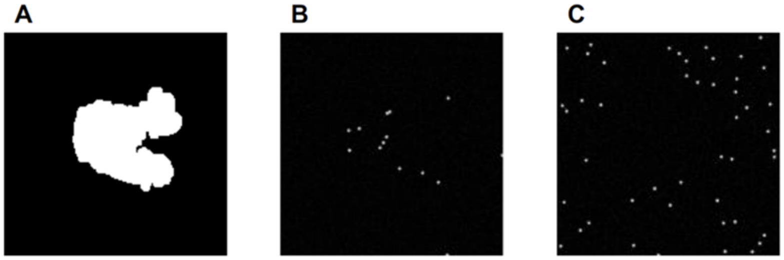

Supplementary Video 4: Simulation of QDots diffusing through a cell contact. (A) One of the cell boarders used to create simulation data. (B) Simulation where QDots start indside the cell contact and freely diffuse outof and within the contact. (C) Simulation where QDots start outside the cell contact and freely diffuse into and outside the contact zone.

	Standard				Dilate				Erode				
Δ(P,Q) (nm)	-11.3	-4.1	7.7	14.9	-11.3	-4.1	7.7	14.9	-11.3	-4.1	7.7	14.9	Sim
Number of attempts to enter	197	191	452	240	155	204	410	247	13	19	92	48	650
Number of attempts to exit	27	44	160	118	159	177	433	322	8	13	62	36	225

Supplementary Table 1: Number of tracks attempting to enter or exit the cell contact and success to enter or exit.

A

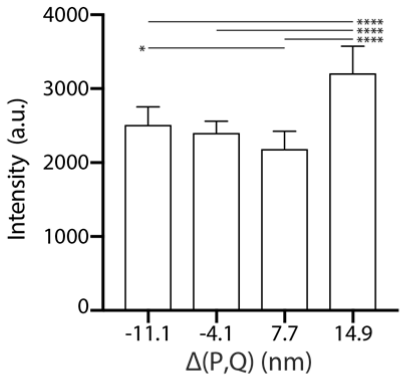

B

Intensity zone	Δ(P,Q) (nm)							
	-11.1		-4.1		7.7		14.9	
	Mean	Std	Mean	Std	Mean	Std	Mean	Std
Outside	2711	283	2981	184	3117	289	3988	418
(1-1.5)	3235	185	3455	197	3558	218	4665	929
(1.5-2)	3862	199	4022	182	4300	214	5428	796
>2	4383	159	4555	40	6050	740	6168	601

Supplementary Figure 1: (A) Mean intensities of protein spacers outside the cell contact. P values were obtained from a one-way ANOVA test. (B) Mean intensities of different intensity zones inside and outside the cell contact. The intensity zone (1-1.5, 1.5-2.0 and more than 2.0 times the outside intensity) are within the cell contact.

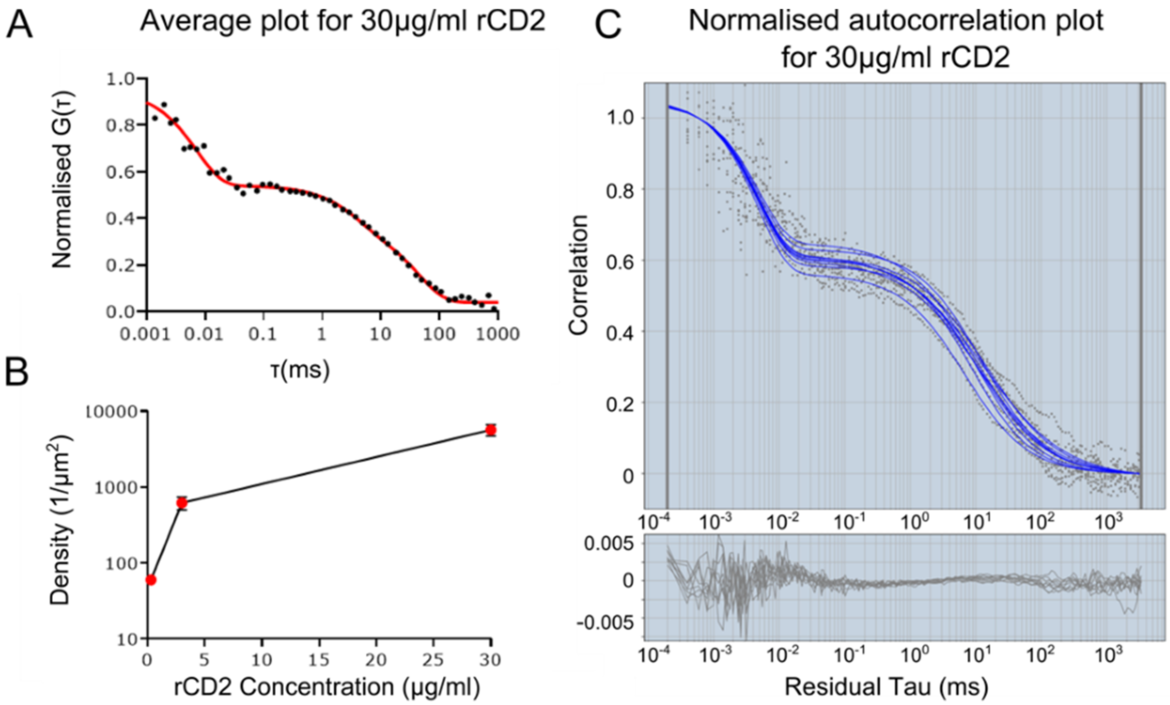

Supplementary Figure 2: Protein density determination using FCS. A) Average plot for 30 µg/ml rCD2. Measurements were performed using underlabelled conditions since FCS curves could not be obtained at a concentration of 30 µg/ml because the sensor was saturated preventing any accurate readings. Instead we used a mixture of labelled and unlabelled rCD2 (10% labelled Alexa 647 and 90% unlabelled). The obtained density was then multiplied by 10 to give the 'true' density reading. Three FCS readings (10 s each) were taken on three bilayers for each concentration of rCD2 tested (9 FCS readings total per concentration). FCS data was analysed using FoCuS-point (https://www.ncbi.nlm.nih.gov/pubmed/26589275). B) Protein density measured at different rCD2 concentrations measured via FCS. C) Normalised autocorrelation for 30 µg/ml rCD2.

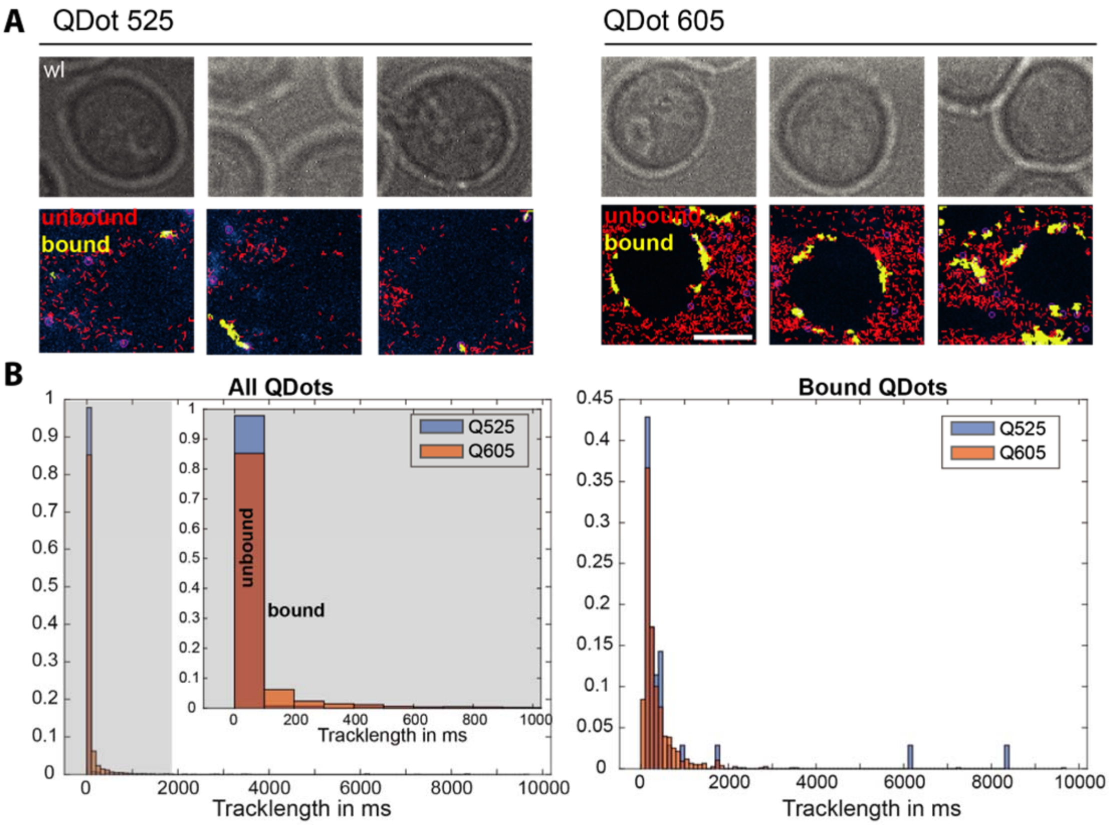

C

	Q525	Q605
**tracks selected as bound** (tracks longer than 5 frames at 20ms frame rate = 100ms)	4.7% ±3.7%	11.4% ±5.1%
**tracks relevant for analysis** (tracks longer than 5 frames at 60ms frame rate = 300ms)	1.8% ±2.1%	4.9% ±2.2%

Supplementary Figure 3: Unspecific binding of QDots to cells is minor. Jurkat rCD48 cells were incubated with QDots at a concentration of 500 nM. Cells were imaged in HILO to observe unspecific, temporary binding of QDots to cells at high frame rates. (A) The upper panel shows the white light and QDot channel with tracks coloured in bound (yellow) vs unbound (red). Scale bar, 5 µm. (B) Histograms of tracklengths of all QDots trackable in the video (All QDots, left). Tracks longer than five frames were classified as bound (right). Data was collected from five cells per condition. (C) Table gives mean ± SD percentage of QDots bound longer than five frames as indication of temporary non-specific interactions (tracks selected as bound). Since analysis rejects tracks shorter than five frames the only relevant tracks are those bound for longer than 300 ms (tracks relevant for analysis).

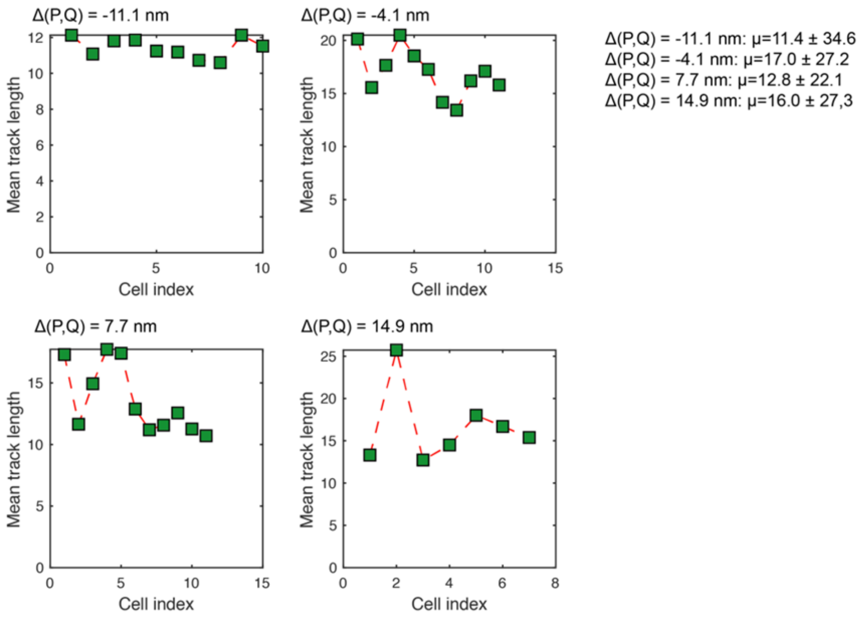

Supplementary Figure 4: Average tracklength in frames for each cell measured. μ states the mean tracklength per condition.

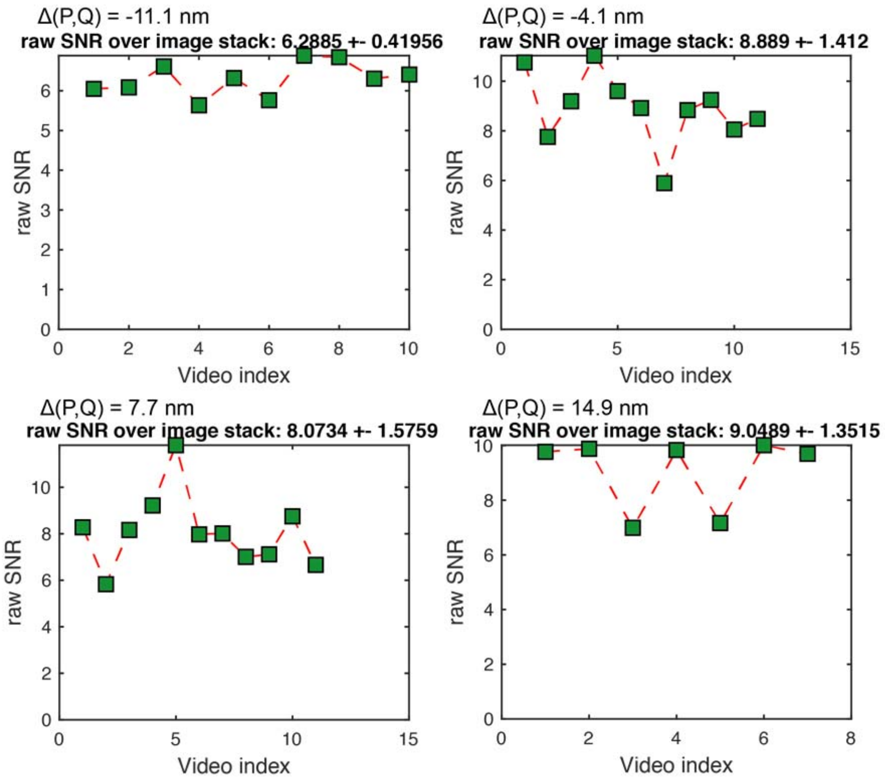

Supplementary Figure 5: Average signal-to-noise-ratio for each cell measured.

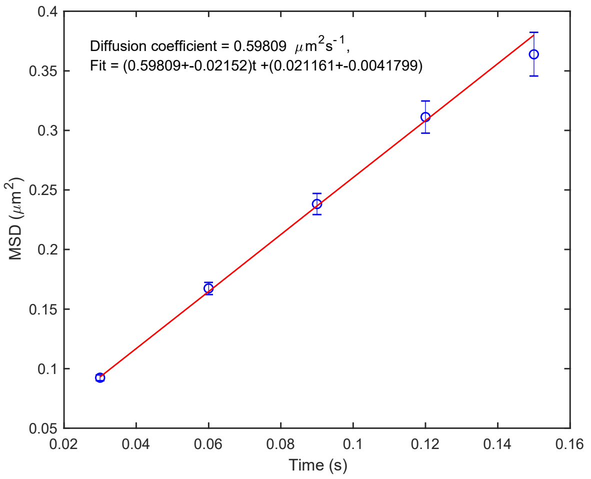

Supplementary Figure 6: MSD plot of QDots (Q605) freely diffusing in bilayers. The MSD curve was fitted using a custom-written Matlab code by Weimann *et al.*(33)

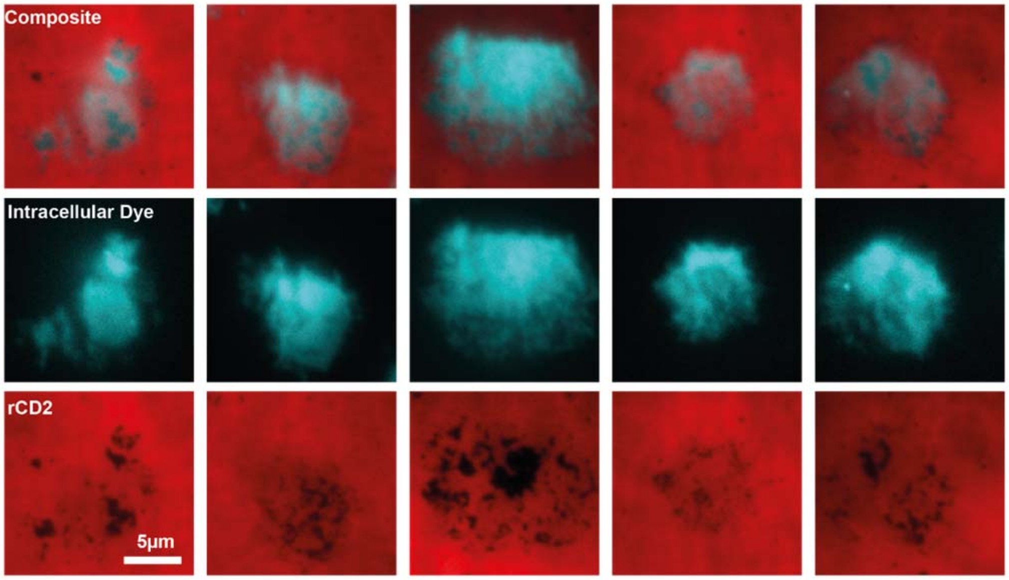

Supplementary Figure 7: Contacts are mediated by ratCD2 and rat CD48 constructs. Expression of rCD48 is necessary for rCD2 accumulation. Jurkat cells which do not express rCD48 were dropped on rCD2 (Alexa647) bilayers. To indicate the position of the cells without leading to membrane dye leakage into the bilayer, cells were labelled intracellularly with Fluo4 dye.

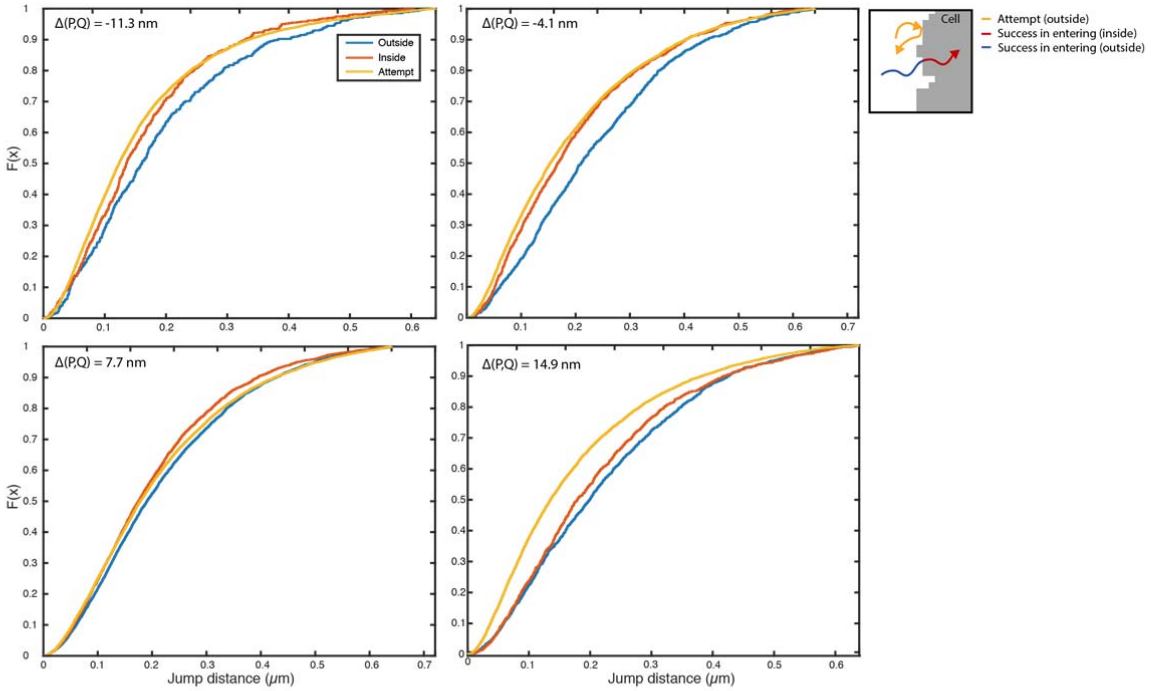

Supplementary Figure 8: JD distributions for different gap conditions for successful tracks, separated out according to being inside or outside the contact. Blue curves correspond to JD values from the outside part of QDot tracks which successfully entered the contact, red curves are the JD values of the tracks inside the contact, after successfully entering the contact. The yellow curves are JD values from tracks attempting but failing to enter the contact.

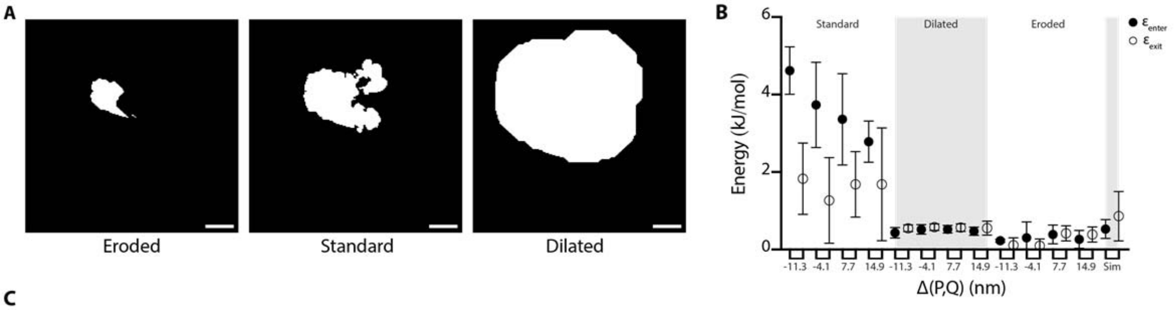

C

		Standard entering				Standard exiting				Dilate entering				Dilate exiting				Erode entering				Erode exiting					
	Δ(P,Q) (nm)	-11.3	-4.1	7.7	14.9	-11.3	-4.1	7.7	14.9	-11.3	-4.1	7.7	14.9	-11.3	-4.1	7.7	14.9	-11.3	-4.1	7.7	14.9	-11.3	-4.1	7.7	14.9	Sim entering	Sim exiting
Standard enteri	-11.3		ns	**	****	****	****	****	****	****	****	****	****	****	****	****	****	****	****	****	****	****	****	****	****	****	****
	-4.1	ns		ns	ns	***	****	****	****	****	****	****	****	****	****	****	****	****	****	****	****	****	****	****	****	****	****
	7.7	**	ns		ns	***	****	****	****	****	****	****	****	****	****	****	****	****	****	****	****	****	****	****	****	****	****
	14.9	****	ns	ns		ns	**	*	ns	****	****	****	****	****	****	****	****	****	****	****	****	****	****	****	****	****	****
Standard exiting	-11.3	****	***	***	ns		ns	ns	ns	**	ns	*	*	*	ns	*	*	**	*	**	**	*	*	**	**	**	ns
	-4.1	****	****	****	**	ns		ns	ns	ns	ns	ns	ns	ns	ns	ns	ns	ns	ns	ns	ns	ns	ns	ns	ns	ns	ns
	7.7	****	****	****	*	ns	ns		ns	***	*	**	**	**	ns	**	*	ns	**	***	***	*	*	**	**	***	ns
	14.9	****	****	****	ns	ns	ns	ns		**	ns	*	*	*	ns	*	ns	ns	*	**	**	*	*	**	*	**	ns
Dilate entering	-11.3	****	****	****	****	**	ns	***	**		ns	ns	ns	ns	ns	ns	ns	ns	ns	ns	ns	ns	ns	ns	ns	ns	ns
	-4.1	****	****	****	****	ns	ns	*	ns	ns		ns	ns	ns	ns	ns	ns	ns	ns	ns	ns	ns	ns	ns	ns	ns	ns
	7.7	****	****	****	****	*	ns	**	*	ns	ns		ns	ns	ns	ns	ns	ns	ns	ns	ns	ns	ns	ns	ns	ns	ns
	14.9	****	****	****	****	*	ns	**	*	ns	ns	ns		ns	ns	ns	ns	ns	ns	ns	ns	ns	ns	ns	ns	ns	ns
Dilate exiting	-11.3	****	****	****	****	*	ns	**	*	ns	ns	ns	ns		ns	ns	ns	ns	ns	ns	ns	ns	ns	ns	ns	ns	ns
	-4.1	****	****	****	****	ns	ns	ns	ns	ns	ns	ns	ns	ns		ns	ns	ns	ns	ns	ns	ns	ns	ns	ns	ns	ns
	7.7	****	****	****	****	*	ns	**	*	ns	ns	ns	ns	ns	ns		ns	ns	ns	ns	ns	ns	ns	ns	ns	ns	ns
	14.9	****	****	****	****	*	ns	*	ns	ns	ns	ns	ns	ns	ns	ns		ns	ns	ns	ns	ns	ns	ns	ns	ns	ns
Erode entering	-11.3	****	****	****	****	**	ns	ns	ns	ns	ns	ns	ns	ns	ns	ns	ns		ns	ns	ns	ns	ns	ns	ns	ns	ns
	-4.1	****	****	****	****	*	ns	**	*	ns	ns	ns	ns	ns	ns	ns	ns			ns	ns	ns	ns	ns	ns	ns	ns
	7.7	****	****	****	****	**	ns	***	**	ns	ns	ns	ns	ns	ns	ns	ns	ns	ns		ns	ns	ns	ns	ns	ns	ns
	14.9	****	****	****	****	**	ns	***	**	ns	ns	ns	ns	ns	ns	ns	ns	ns	ns	ns		ns	ns	ns	ns	ns	ns
Erode exiting	-11.3	****	****	****	****	*	ns	*	*	ns	ns	ns	ns	ns	ns	ns	ns	ns	ns	ns	ns		ns	ns	ns	ns	ns
	-4.1	****	****	****	****	*	ns	*	*	ns	ns	ns	ns	ns	ns	ns	ns	ns	ns	ns	ns	ns		ns	ns	ns	ns
	7.7	****	****	****	****	**	ns	**	**	ns	ns	ns	ns	ns	ns	ns	ns	ns	ns	ns	ns	ns	ns		ns	ns	ns
	14.9	****	****	****	****	**	ns	**	*	ns	ns	ns	ns	ns	ns	ns	ns	ns	ns	ns	ns	ns	ns	ns		ns	ns
	Sim entering	****	****	****	****	**	ns	***	**	ns	ns	ns	ns	ns	ns	ns	ns	ns	ns	ns	ns	ns	ns	ns	ns		ns
	Sim exiting	****	****	****	****	ns	ns	ns	ns	ns	ns	ns	ns	ns	ns	ns	ns	ns	ns	ns	ns	ns	ns	ns	ns	ns	

Supplementary Figure 9: Energy penalties for experimental, eroded and dilated contact borders. (A) Contact masks used to test success rate of QDots entering and exiting an 'artificial' contact, scale bars 5 µm. (B) Energy penalties for entering and exiting eroded, real and dilated contacts. C) Table of p-Values comparing all gap conditions with all eroded, dilated, simulated and experimental contacts.

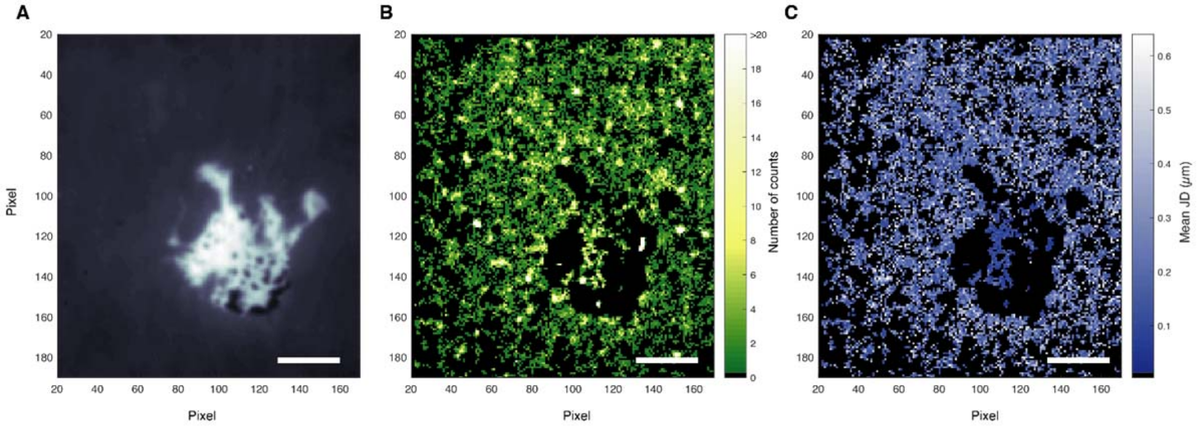

Supplementary Figure 10: (A) Fluorescence image of a cell binding to CD2CD45 in a SLB. Scale bar, 5 μm. (B) Map showing the number of counts per pixel of a CD2CD45 / QD605 contact. Mean number of counts per pixel μ=5.2 ±11.9. (C) Corresponding map of jump distances.

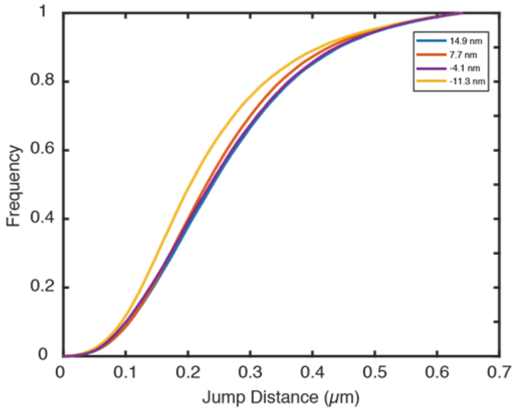

Supplementary Figure 11: ECDFs of overall jump distances in track image inside the contacts.

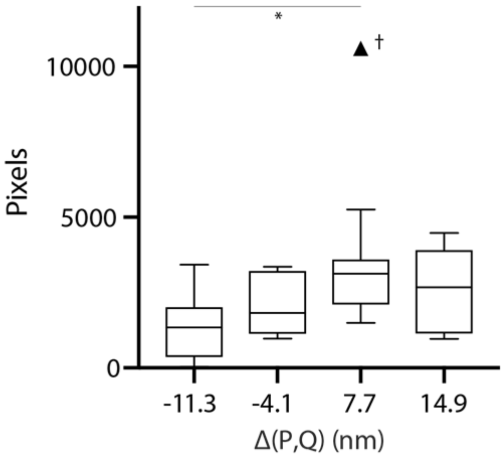

Supplementary Figure 12: Contact size distribution in pixel for each condition. A one-way Anova comparing the means of each condition did not show a significant difference, other than between Δ(P,Q) = -11.3 nm and 7.7 nm. †This data point shows a field of view containing two cells.

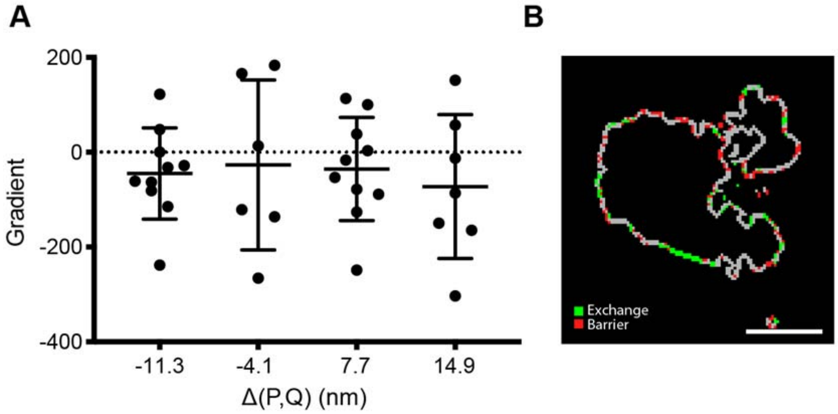

Supplementary Figure 13: Gradient coefficient for each linear fit for the success rate plotted against the intensity at that pixel for all border pixels. The success rate was weighted to the number of attempts to enter at the position of the pixel. B) Representative border image of a rCD2rCD45 Contact probed with a QDot605 spacer. Pixels in the border where no exchange occurred are marked in white, pixels where probes where reflected off are marked red and pixels where probes exchanged over the barriers are marked green. Scale bar 5 µm.

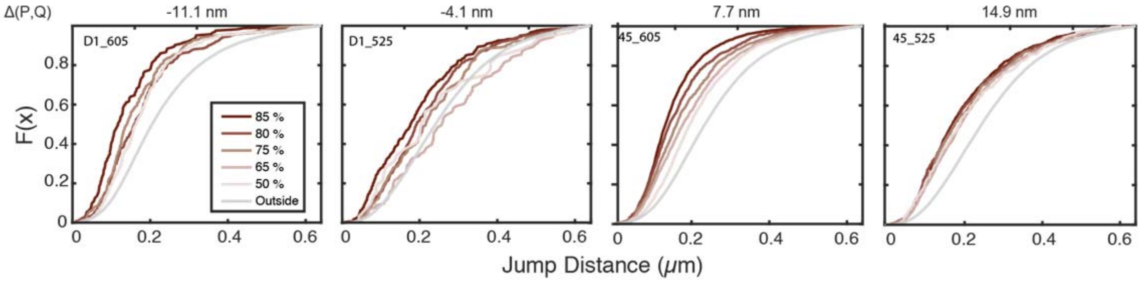

Supplementary Fig 14: ECDFs of JD populations separated via intensity zones for different gap conditions

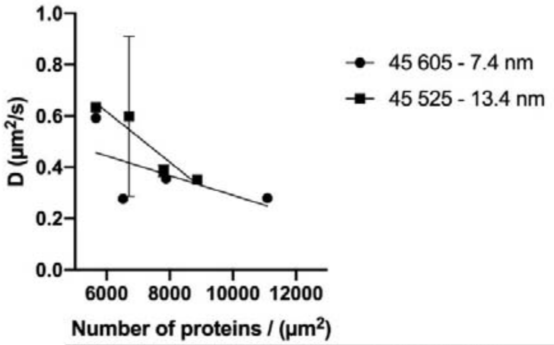

	45 605, 7.7 nm	45 525, 14.9 nm
Slope	-3.836e-005	-9.856e-005
Y-intercept	0.6747	1.208

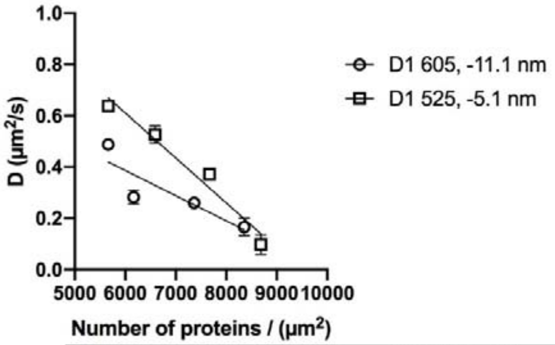

	D1 605, -11.1 nm	D1 525, -5.1 nm
Slope	-9.820e-005	-0.0001754
Y-intercept	0.9759	1.663

Supplementary Figure 15: Results for linear fit of diffusion constants in areas of protein number.
