# Supplementary material for: The Costs of Close Contacts: Visualizing the Energy Landscape of Cell Contacts at the Nanoscale
Source: Biophys J. 2020 Jan 28;118(6):1261–9. doi: 10.1016/j.bpj.2020.01.019 (PMC7091464; doi:10.1016/j.bpj.2020.01.019)
Supplement: Document S2. Article plus Supporting Material [file mmc6.pdf]

Biophysical Journal
Article

Biophysical Society

The Costs of Close Contacts: Visualizing the Energy Landscape of Cell Contacts at the Nanoscale

Klara Kulenkampff,[1] Anna H. Lippert,[1] James McColl,[1] Ana Mafalda Santos,[2] Aleks Ponjavic,[1] Edward Jenkins,[2] Jane Humphrey,[1] Alexander Winkel,[3] Kristian Franze,[3] Steven F. Lee,[1] Simon J. Davis,[2] and David Klenerman[1,*]
[1]Department of Chemistry, University of Cambridge, Cambridge, United Kingdom; [2]Radcliffe Department of Medicine and MRC Human Immunology Unit, John Radcliffe Hospital, University of Oxford, Oxford, United Kingdom; and [3]Department of Physiology, Development and Neuroscience, University of Cambridge, Cambridge, United Kingdom

ABSTRACT Cell-cell contacts often underpin signaling between cells. For immunology, the binding of a T cell receptor to an antigen-presenting pMHC initiates downstream signaling and an immune response. Although this contact is mediated by proteins on both cells creating interfaces with gap sizes typically around 14 nm, many, often contradictory observations have been made regarding the influence of the contact on parameters such as the binding kinetics, spatial distribution, and diffusion of signaling proteins within the contact. Understanding the basic physical constraints on probes inside this crowded environment will help inform studies on binding kinetics and dynamics of signaling of relevant proteins in the synapse. By tracking quantum dots of different dimensions for extended periods of time, we have shown that it is possible to obtain the probability of a molecule entering the contact, the change in its diffusion upon entry, and the impact of spatial heterogeneity of adhesion protein density in the contact. By analyzing the contacts formed by a T cell interacting with adhesion proteins anchored to a supported lipid bilayer, we find that probes are excluded from contact entry in a size-dependent manner for gap-to-probe differences of 4.1 nm. We also observed probes being trapped inside the contact and a decrease in diffusion of up to 85% in dense adhesion protein contacts. This approach provides new, to our knowledge, insights into the nature of cell-cell contacts, revealing that cell contacts are highly heterogeneous because of topography- and protein-density-related processes. These effects are likely to profoundly influence signaling between cells.

SIGNIFICANCE The spatial distribution and diffusion of proteins have been shown to be important for various signaling machineries. As such, size-dependent reorganization of proteins in the immune cell contact has been shown to affect activation of immune cells. Because these studies relied on bulk measurements to investigate protein exclusion, small-scale topographical changes and protein dynamics could not be evaluated. However, recent studies show that T cell activation is mediated by nanoscale structures. In our study, we use molecular probes of various sizes to investigate the energy landscape of single molecules in a cell contact. This provides additional information and insights that cannot be determined by performing bulk experiments alone.

INTRODUCTION

Cells in multicellular organisms are continually in contact with other cells and signal through a variety of mechanisms (1–3). Juxtacrine, or cell-cell signaling, is a process wherein two cells form a sustained contact, allowing molecules on the cell surfaces to interact and exchange. In the case of lymphocytes, fragments of pathogens presented by antigen-presenting cells engage the T cell receptor (TCR) across these contacts, leading to signaling, T cell activation, and eventually an immune response. Activation results in the large-scale spatial reorganization of other important membrane proteins, including signaling and adhesion proteins, into a structure called the immunological synapse (4).

Although we understand very little about the mechanisms that lead to this restructuring, the dimensions and steric properties of surface proteins in the contact are thought to be important for their spatial arrangement (5–8). In this context, it has been suggested that passive rearrangements alone, in

Submitted July 9, 2019, and accepted for publication January 16, 2020.
*Correspondence: dk10012@cam.ac.uk
K. Kulenkampff and A. H. Lippert contributed equally to this work.
Editor: Claudia Steinem.
https://doi.org/10.1016/j.bpj.2020.01.019
© 2020 Biophysical Society.
This is an open access article under the CC BY license (http://creativecommons.org/licenses/by/4.0/).

which the contact gap between the two cells is set by adhesion molecules matching the dimensions of the TCR-pMHC complex (∼14 nm), could explain TCR triggering (9–13). The extracellular domains of other signaling proteins, such as the inhibitory phosphatase CD45, extend well beyond this distance. In the contact, CD45 would experience size-dependent exclusion, and the lack of inhibition at the contact is proposed to contribute to signaling (9,14–16). The contact, however, is a very complex, densely populated environment where few TCR-pMHC interactions are sufficient to initiate an immune response (17–19). Here, single-molecule diffusion studies showed that TCR-pMHC binding and dwell time are important for T cell signaling and antigen discrimination (20,21). Therefore, the behavior of single proteins inside this contact is highly relevant, and the understanding of how the contact environment restricts access of inhibiting, larger proteins is highly desirable. However, the diffusion of proteins in the contact is influenced by many factors (20–22) such as protein-protein interactions (23) and putative lipid rafts (24), as well as the cytoskeleton (23,25,26) and confinement due to crowding (27). Apart from steric hindrance, a contact between two membranes could lead to increased binding due to closer proximity and could exert forces on receptors (19). Various studies on protein and ligand dynamics inside this contact environment have led to contradictory observations concerning the importance of bond lifetimes and protein diffusion for cell activation (15–17).

To understand the relevance of steric exclusion and the contribution of physical restrictions on protein diffusion in contacts, it is important to develop methods for analyzing how the contact gap size affects protein behavior at the single-molecule level. Here, using a probe of known dimensions, we have been able to study the effect of steric hindrance and crowding experienced by proteins at the contact. Previous studies used small fluorescent molecules, including sugars (28), fluorescent proteins (27), and quantum dots (QDots) (29,30), to probe cell-cell, cell-surface, or membrane-membrane contacts in bulk experiments. They identified a size threshold leading to partial-to-complete clearing of the immune synapse or contact for particles 2–10 nm larger than the expected contact. Although these experiments yielded important insights into the general size-excluding properties of contacts, it has remained unclear how contact topography, particle exchange across contact boundaries, and energy penalties for entering and exiting the contact influence protein organization at cell contacts.

MATERIALS AND METHODS

Cell culture

The Jurkat rCD48 cell line was generated via lentivirus transfections under the pHR vector. Expression levels were measured to be around 30,000 proteins per cell. T cells were cultured in phenol-red-free RPMI supplemented with 10% fetal calf serum, 1% HEPES buffer, 1% sodium pyruvate, and 1% penicillin-streptomycin.

Supported lipid bilayer formation

Supported lipid bilayers (SLBs) were prepared according to previous protocols (31). Glass cover slides (VWR, Radnor, PA) were cleaned for 1 h using piranha solution (3:1 sulfuric acid/hydrogen peroxide). After rinsing with MilliQ water, the slides were plasma cleaned for 30 min, and a silicon well (Grace Bio-Labs, Bend, OR) was attached to each slide. Previously prepared small unilamellar vesicle solution was added to each phosphate-buffered saline (PBS)-filled silicone well and incubated for 30 min. The vesicle solution consisted of 1 mg/mL of 95% POPC, 4.999% DGS-NTA(Ni), and 0.001% Biotinyl-Cap-PE (all Avanti Polar Lipids, Alabaster, AL). After incubating for 30 min, the wells were washed three times with PBS solution, and the protein solution was added at 30 μg/mL. Purified protein spacers were provided by the Davis lab in Oxford. The proteins used were either rCD2.D1, a truncated protein with domain 1 of rCD2, or a chimeric protein that comprises the extracellular portion of rCD2 plus rCD45 (rCD2rCD45). Each of the proteins was labeled with Alexa Fluor 647 (Thermo Fisher Scientific, Waltham, MA). After 1 h incubation at room temperature, the wells were washed three times with PBS, and cells were added. All bilayer conditions showed similar protein fluorescence intensities, with variations of maximal 11% standard deviation (SD) within one condition and mean variations of maximal 15% SD between the conditions (see Fig. S1). Protein densities in the bilayer were measured via fluorescence correlation spectroscopy (FCS) and found to be 5660 $\pm$ 860 proteins per μm^2 (see Fig. S2). Measurements were performed on underlabeled conditions (10% labeled Alexa 647 rCD2 and 90% unlabeled rCD2) because FCS curves could not be obtained at a concentration of 30 μg/mL because the sensor was saturated, preventing any accurate readings. The obtained density was then multiplied by 10 to give the “true” density reading. FCS data were analyzed using FoCuS-point (32).

Sample preparation

Streptavidin-coated QDots (QDot 525 Streptavidin Conjugate and QDot 605 Streptavidin Conjugate; both Thermo Fisher Scientific) were suspended onto the bilayer at a concentration of 50 nM in PBS. The QDots were left for 5 min before washing two times with PBS to remove QDots that had not bound to biotinylated headgroups. To quantifying unspecific short-term binding of QDots to cells in solution, cells were imaged around the midplane in a highly inclined and laminated optical sheet in a bath of QDots (500 nM) at 10 ms exposure (Fig. S3; Video S1). Assuming every tracked QDot has had the chance to interact with the cell during the 20 s of acquisition, because the particles explore a much larger volume (3600 μm^3 or 4100 μm^3) than the acquisition volume (46 μm^3 or 40 μm^3, defined by the depth of field of the objective at 525 or 605 nm emission and the acquisition area), we only observe 1% (QD525) and 4.9% (QD605) of nonspecific temporary binding. This, however, is an upper limit because many other QDots in solution are faster, which leads to blurring; therefore, they would not be tracked. Theoretically, the lower limit can be estimated using Fick’s laws to calculate the adsorption rate $r = 0.5\,Ac_0\sqrt{\pi D/t}$ of a dilute fluid at concentration c_0 to a surface A. This yields around 30,000 collisions during the acquisition and therefore a lower limit for the nonspecific binding rate of 0.03%.

Data acquisition

Before imaging, the cells were washed in PBS and then added to the rCD2 constructs and QDot-containing bilayer (preparation described above). Imaging was performed using a custom-built total internal reflection (TIRF) setup using a 100× Apo TIRF, numerical aperture 1.49 objective (Nikon, Tokyo, Japan), creating a TIRF illumination at the glass water interface. Fluorescence was recorded through a beam splitting system (Dual-View; BioVision Technologies, Exton, PA) using a dichroic mirror and filters (for 488 or 633 emission, FF605-Di02 (dichroic; Photometrics, Tucson, AZ), FF03-525/50-25 (filter, 488 emission), and BLP01-635R-25 (filter,

633 emission), all Semrock, Rochester, NY). 1000 frames per experiment were acquired with an exposure time of 30 ms, yielding an overall recording time of 1 min. The camera (Cascade II; Photometrics) and shutter (SH05; Thorlabs, Newton, NJ) were operated using Micromanager (Vale Lab, University of California-San Francisco, San Francisco, CA). The two Dual-View channels were aligned using TetraSpec Microspheres (0.1 μm, fluorescent blue, green, orange, and dark red; Thermo Fisher Scientific) routinely to a precision of ~120 nm.

Data analysis: Single-particle tracking

Single-particle tracking was performed using a custom-written MATLAB (The MathWorks, Natick, MA) code (33), yielding diffusion behavior and coefficients from jump-distance (JD) distributions of the QDots. Here, only tracks longer than five frames were used, with mean track lengths of 14.3 ± 2.3 and a signal-to-noise ratio of 8.0 ± 1.1 (Figs. S4 and S5).

Exclusion analysis

To analyze the size-dependent exclusion, contact masks were defined by thresholding the gradient of the fluorescence of protein spacers (threshold set to 0.2 times the maximal gradient of an image) using a custom-written MATLAB code. Furthermore, a small size threshold was applied to exclude bilayer inhomogeneities. These masks were used to distinguish between QDots inside and outside the contact by performing peak find on the first frame of every experiment to calculate the ratio of density inside/outside the contact (using Fiji, an open-source platform for biological image analysis).

Energy penalty

We used the contact masks to divide tracks and jumps into four categories: 1) tracks that are outside the contact mask and stay outside the contact mask, 2) tracks that are outside the contact mask and end inside the contact mask, 3) tracks that start inside the contact mask and stay inside, or 4) tracks that start inside the contact mask and end outside the contact mask. To avoid bias toward unsuccessful entries or exits of QDots, we analyzed only tracks that were recorded for at least five frames after touching the cell border. Hence, we could analyze parameters such as JD in the corresponding location category, number of attempts of a QDot to enter cell gap, and number of successful entries into or exits out of the gap.

Using those success rates, we could calculate energy penalties, ε_i, for entering and exiting the contact via the Boltzmann distribution, $p_i = \exp(-\varepsilon_i/kT)$, with $p_i = (n_{success}/n_{attempts})$ to calculate ε_{enter} or ε_{exit}.

Size- and density-dependent slowdown

Using the track ensemble, we created a map of the average speed per pixel of diffusing QDots. The track ensemble was overlaid with a pixel grid corresponding to the original pixel size of the recorded image to determine average JD at the corresponding pixel position. These were displayed with scaled colors corresponding to the magnitude of the average JD. By fitting the JD distribution jumps occurring inside or outside the contact, we could determine the diffusion coefficient inside and outside the contact and across different intensity zones (corresponding to different protein densities). JD distributions were fitted to the probability distribution function $P(r^2, \Delta t) = 1 - \exp(r^2/r_0^2(t))$, where $r_0^2(t) = 4Dt + 4\sigma^2$, of a particle with a diffusion coefficient D and localization precision σ to be within the radius r of a shell at a time Δt (34) using a MATLAB built-in nonlinear least-squares fitting function, yielding r_0^2. The cumulative distribution and the resulting fit were then plotted over $\sqrt{r^2} = r$. Error bars are acquired via bootstrapping of the JD distribution. As Weimann et al. (33) showed via simulations, at the localization precision achieved in our experiments (see Fig. S6), errors due to localization precision were found to be smaller than errors found via bootstrapping of the JD distribution.

Statistical testing

One-way analysis of variance and post hoc tests were used as noted (Prism 8; GraphPad, San Diego, CA) using a significance level of $\alpha < 0.05$ for analysis of variance and for post hoc tests. The Tukey-Kramer method was applied to evaluate p values in post hoc tests.

RESULTS AND DISCUSSION

QDots of various sizes experience size-dependent exclusion

Exclusion of molecules from cell-cell contacts has been proposed as a key factor during lymphocyte signaling (9,10), and although there is evidence accumulating to support this idea (35–37), it remains contentious. We sought to determine whether large, surface-anchored molecules (represented by QDots) would be excluded from contacts by studying the size-dependent exclusion of the QDot probes.

We used SLBs presenting "spacer" adhesion proteins of various sizes and QDots of known diameter freely diffusing in the bilayer as probes (see Fig. S6; Videos S2 and S3). To control QDot density, biotinylated lipids were incorporated into the SLBs, and the spacer proteins were coupled via nickel-chelating lipids. We controlled cell-bilayer gap sizes using modified versions of the adhesion protein rat CD2 (CD2) as spacer protein (Fig. 1 *A*). CD2 has two immunoglobulin superfamily domains in its extracellular region, stacked on top of one another, with the membrane distal domain binding rat CD48, which we expressed at the surface of the human Jurkat T cells. Here, Jurkat cells that did not express rat CD48 failed to accumulate rat CD2 (see Fig. S7); therefore, any binding not mediated by rat CD2 and rat CD28 can be neglected. The distance spanned by this complex is ~13.4 nm (14). To create small gaps, we used a short, truncated version of CD2 comprised of only the ligand binding domain (CD2d1). For larger gaps, we used the extracellular region of rat CD2 and the folded part of the extracellular domain of human CD45 (CD2CD45), a large receptor-type phosphatase (Fig. 1 *A*). The CD2d1:CD48 complex forms a gap predicted to be 9.4 nm and CD2CD45:CD48 a 34.4 nm gap (14,38). To avoid any direct signaling effects through CD48 binding, the cells expressed a truncated form of the receptor lacking a cytoplasmic region. The cells were then dropped onto an SLB containing QDot and CD2 variants, allowing formation of "spacer" complexes. Whereas the bound QDots diffuse freely with a diffusion coefficient of around 0.6 $\mu m^2\ s^{-1}$ (see Fig. S6; Videos S2 and S3), the cells accumulated CD2 and formed contacts. To probe the effect of steric hindrance on probes of known height, we used QDots of two sizes, Q605 and Q525, which have hydrodynamic diameters of 21.2 nm (29) and 14 nm (Thermo Fisher Scientific,

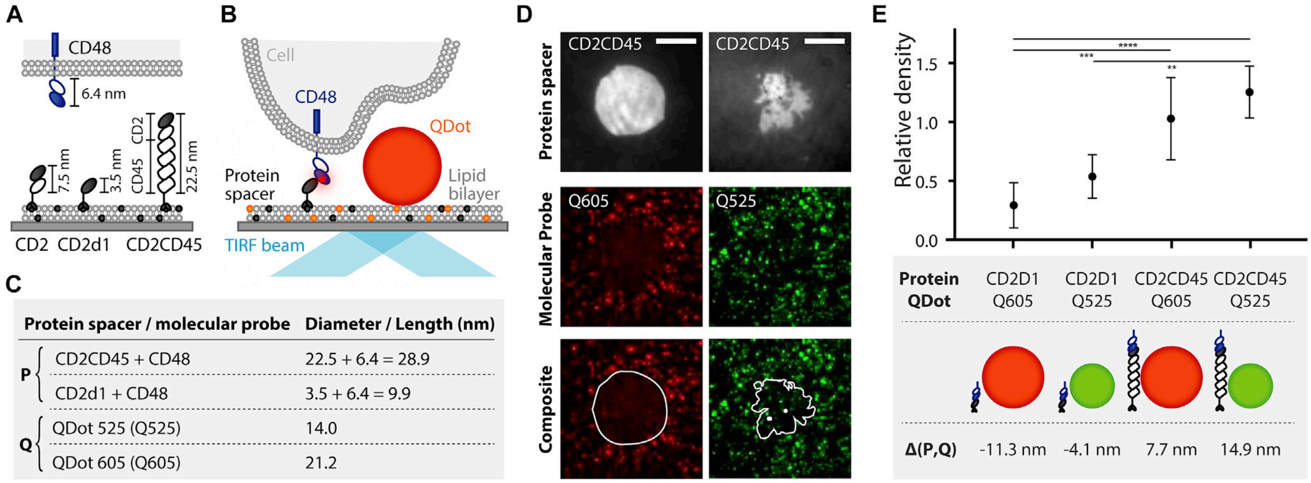

FIGURE 1 Gap-size-dependent QDot exclusion. (*A* and *B*) Schematic representation (*A*) of the protein spacers and their ligand (CD48: 6.4 nm) used in the experiments (CD2d1: 3.5 nm or CD2CD45: 22.5 nm) and the experimental configuration (*B*): streptavidin-conjugated QDots (Q525 or Q605) were seeded onto a supported lipid bilayer (SLB) containing biotinylated headgroups (*orange*). Cells form a contact with the SLB by binding protein spacers (P); the contact is anchored to the SLB through histidine tags on the protein-binding nickel-chelating lipids (*dark grey*). Spacers fluorescently labeled with Alexa 647 are binding CD48 on the cell, creating gaps of different sizes. (*C*) The length of each protein spacer and the hydrodynamic diameter of QDots used in SLB experiments in this work are presented in the table. (*D*) TIRF images and composites of the contact formed by CD2CD45 (*top*) and QDots (*middle*) in the SLB (*right*: QDot605 (*red*), *left*: QDot525 (*green*)) are shown. Scale bars, 5 μm. (*E*) QDot density inside the cell-bilayer contact is compared to the density outside the contact to gain relative density. Δ(P, Q) = P − Q is the difference between QDot diameter and spacer-protein-induced gap size. Number of cells (n) and standard deviation (SD) for each condition: Δ(P, Q) = −11.3 nm, n = 6, SD = ±59.7%; Δ(P, Q) = −4.1 nm, n = 3, SD = ±28.0%; Δ(P,Q) = 7.7 nm, n = 6, SD = ±31.0%; and Δ(P, Q) = 14.9 nm, n = 6, SD = ±16.0%. Data were analyzed using a one-way analysis of variance (ANOVA). $^{**}p < 0.01$, $^{***}p < 0.001$, $^{****}p < 0.0001$.

Bremen, Germany, personal communication), respectively (Fig. 1 *B*). We termed the difference between the gap created by the spacer complex (P) and the QDot diameter (Q), Δ(P, Q) = P − Q. This value ranged from 11.3 to 14.9 nm, and we expected to observe size-dependent exclusion for negative Δ(P, Q) values (Fig. 1 *B*).

To examine the extent of size-dependent exclusion, we determined the QDot density inside relative to outside the contact (Fig. 1, *C* and *D*). We observed a lower QDot density beneath the contact region, relative to the density of QDots in the surrounding area, even when the height difference was as small as −4.1 nm (Fig. 1 *E*). The relative density was 29.3 ± 17.5% for the large and 53.7 ± 15.1% for the small QDots in contacts with the smallest gap, giving rise to size-dependent exclusion (exclusion = 1 − relative density) of 46.3% (Δ(P, Q) = −4.1 nm) and 70.7% (Δ(P, Q) = −11.3 nm). Positive Δ(P, Q) values did not result in observable exclusion effects.

Bulk fluorescence imaging of the distribution of large and small molecules at cell contacts can convey the impression that it is an "all or nothing" effect—proteins larger than the gap are wholly excluded from contacts, whereas proteins smaller than the gap can gain access—but single-molecule measurements suggest otherwise (37,39). Our single-molecule data confirmed that even molecules twice the size of the gap were not wholly excluded (Fig. 1 *E*). We could also observe exclusion effects in contacts 4.1 nm smaller than the QDot (Fig. 1 *E*), which is in good agreement with bulk size-exclusion measurements in cell-cell contacts (30) and model membrane systems (27). A difference of 3.2 nm was enough to exclude QDots in other studies (29). This suggests that at cell contacts, size exclusion very likely fine-tunes the distribution of proteins rather than having a purely "all or nothing" size-exclusion effect.

QDots are size-dependently restricted from contact entry

Having established that QDots are gap-size-dependently excluded, we next studied the entry and exit of QDots from the contact. By tracking single QDots at the border of the contact, we classify the trajectories as entering, exiting, and deflected at the contact border (Fig. 2, *A–C*). Tracks starting outside the contact and ending inside it were classified as "entering"; tracks exhibiting the opposite behavior were taken to be "exiting." "Deflected" tracks were classified as tracks starting outside, having one or more localizations on the contact border, and ending outside the contact. Using the rate of entries or exits to attempts, we calculated the energy penalty for entering (ε_{enter}) and exiting (ε_{exit}), assuming a Boltzmann distribution (see Materials and Methods; Fig. 2 *D*). For QDots exiting the contact region, ε_{exit} is comparable for all Δ(P, Q) (Fig. 2 *E*). Conversely, for QDots entering the contact, ε_{enter} is Δ(P, Q) dependent, with increasing ε_{enter} for decreasing gap-to-probe sizes (Fig. 2 *E*). We observe a significantly larger ε_{enter}

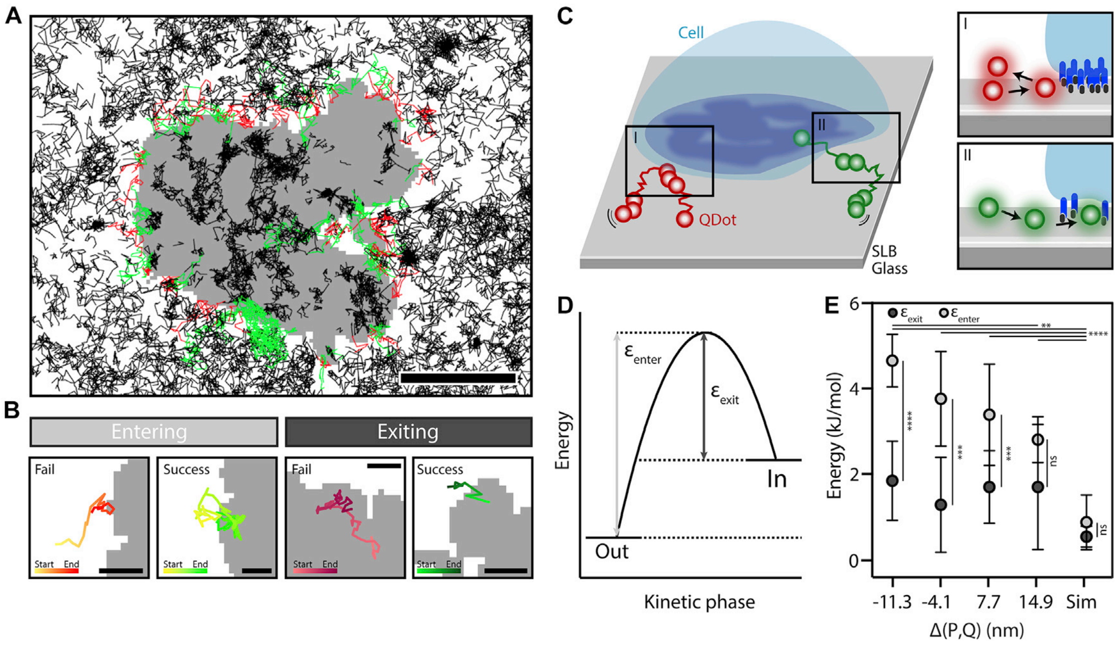

FIGURE 2 Energy penalty of contact entry or exit for QDots. (*A*) Tracks are marked depending on their success in entering the contact (*black*: tracks not attempting to enter or exit the contact, *green*: success in entering the contact, *red*: failure to enter the contact). Scale bars, 5 μm. (*B*) Examples of tracks failing and succeeding to enter (*left*) or exit (*right*) the contact are shown. Scale bars, 1 μm. (*C*) A schematic illustration of QDots attempting to enter the cell contact ((I) fail: *red*, (II) success: *green*) is given. (*D*) The energy landscape for entering or exiting the contact is shown. (*E*) The energy penalty for entering (ε_{enter}) and exiting (ε_{exit}) for different gap-size conditions calculated via success and failed attempts is shown, assuming a Boltzmann distribution (mean $\pm$ SD). The p values were obtained from a one-way ANOVA test and are shown for the success rate in entering. *$p < 0.1$, **$p < 0.01$, ****$p < 0.0001$. The p values above the graphs are energy penalties for entering QDots and below for exiting QDots. Sim shows results for a simulation with 20 repeats and two different contacts. In the simulation, energy penalties are calculated for tracks starting outside and entering the contact (ε_{enter}) and tracks starting inside the contact and exiting (ε_{exit}). Number of cells (n) and SD for each condition: Δ(P, Q) = 14.9 nm, n = 7, SD (enter) = 23.1%, SD (exit) = 66.1%; Δ(P, Q) = 7.7 nm, n = 10, SD (enter) = 40.1%, SD (exit) = 46.4%; Δ(P, Q) = -11.3 nm, n = 8, SD (enter) = 12.4%, SD (exit) = 45.0%; and Δ(P, Q) = 4.1 nm, n = 5, SD(enter)=27.7%, SD(exit)=77.2%. Find all number of attempts, successes, and failures in Table S1.

than ε_{exit} for negative Δ(P, Q) and unexpectedly also for Δ(P, Q) = 7.7 nm. To confirm this result, we simulated particles with starting positions inside the contact using contacts from the acquired data set (see Video S4). We allowed free Brownian diffusion with density and diffusion coefficients adjusted to the experimental data and allowed particles to diffuse freely over the contact. Here, diffusing particles enter and exit the contact without any restrictions, which is shown by the low energy penalties compared to the experimental data. Although the simulations confirm that contact entry is restricted even for positive Δ(P, Q), these values also suggest that QDots exiting the contact also experience a restriction, which is gap-size independent. The spread of ε_{exit} for the experimental data could be an effect of varying protein crowding, which might inhibit exit of proteins from the contact. In addition, by comparing the instantaneous velocity (i.e., JD distribution of QDots that failed to enter) with that of QDots successfully entering the contact, we observed a shift toward lower values for contact gaps with negative Δ(P, Q), also suggesting that there is an energy penalty for contact entry (Fig. S8).

To further verify the increased entering energy penalty for QDots larger than the contact size, we compared ε_{enter} for the measured contact barrier with an area surrounding the contact (dilated mask) in the bilayer (see Fig. S9), where we would not expect entry restrictions. The result confirmed that the observed energy penalty is due to the contact edges acting as a barrier because there is no entry restriction in the dilated masks compared to the contact (see Fig. S9).

QDot slowdown in contacts is independent of size

After having established the contact edge as a barrier, we now focus on the heterogeneity of the contact. Here, areas of reduced velocity were located by studying the behavior of QDots inside the cell contact, measuring the instantaneous velocity or JD of the probes between frames. The steric hindrance landscape was visualized using a map of the average JD of QDots for each pixel (Fig. 3 *A*). We observed a reduction in JD for tracks inside the cell contact, implying steric hindrance, restricted movement, or reduced mobility in the contact. By fitting the JD distribution, we found diffusion coefficients (D) between 0.49 $\pm$ 0.05 and 0.83 $\pm$ 0.03 $\mu m^2/s$ (mean $\pm$ SD) for QDots diffusing outside the cell contact (Figs. 3 *B* and 4 *C*). These values are in agreement with reported lipid diffusion in

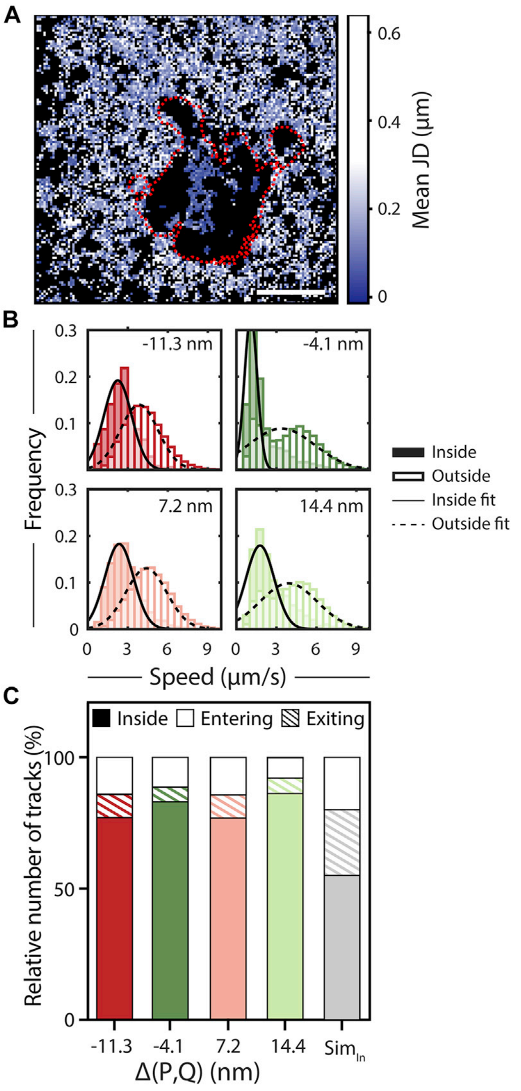

FIGURE 3 Gap-size-dependent slowdown of QDots. (*A*) Jump-distance (JD) map of a cell-bilayer contact. The image shows a representative cell of CD2CD45 spacer proteins used with Q605 diffusing in the SLB. The red dashed line represents the outline of the cell contact. Scale bars, 5 μm. See Fig. S10 for a map of counts per pixels. (*B*) Average speed of QDots inside and outside contact in μm/s is shown. Error bars represent mean $\pm$ SD. Sim shows results for a simulation with 20 repeats and two different contact masks. Tracks were either starting inside (Sim_{in}) or outside the contact. Number of cells n per experiment: Δ(P, Q) = -11.3 nm, n = 10; Δ(P, Q) = -4.1, n = 6; Δ(P, Q) = 7.7 nm, n = 10; Δ(P, Q) = 14.9 nm, n = 7. (*C*) Number of tracks entering (*open*) and exiting (*shaded*) the cell gap in comparison with tracks, which are already inside the cell contact (*solid*).

bilayers, which ranges from 1 to 4 $\mu m^2\ s^{-1}$ (40). The JD distribution of QDots inside the contacts was clearly reduced (Figs. 3 *B* and S8) but did not reveal an accumulation of slow JD indicative of local trapping. Interestingly, the mean velocity inside the contact was constant over all gap-size conditions assessed (Fig. 3 *B*), although we saw a slight shift toward lower velocities in the JD distributions for negative Δ(P, Q) (Fig. S11).

Overall, we observed a trapping effect for all probes that was gap-size independent. We found that 80% of QDots do not leave the contact over the time of acquisition, which is significantly different from simulations (50% remain) (Fig. 3 *C*). This difference between simulation and experiment reflects the larger energy penalty ε_{exit} in experimental data compared to the simulations, which can be explained by the complete absence of protein crowding effects in the simulation data. In experimental data, probes in the contact might experience crowding and trapping effects, hindering their exit gap-size independently. Here, molecules could become trapped because as the cell-bilayer contact forms and as the contact grows, crowding could increase, resulting in it being more difficult for probes to exit the contact. Assuming similar restrictions hold for proteins of comparable size, the contact is a very exclusive environment with exchange limited to ~20%. If we extrapolate these to signaling molecules, this size-independent trapping could lead to a prolonged residence time inside the contact, exposing them, in the case of T cell synapses, to increased kinase/phosphatase ratios.

Although we did not see an overall effect of gap size on QDot velocity, the data clearly revealed a reduction of mean velocity inside compared to outside the contact (Fig. 3 *B*). Interestingly, the reduction in mean velocity was independent of the protein spacer size, suggesting that the molecules were hindered in the contact because of effects such as crowding. The observed differences in energy penalties are not a result of differences in contact areas because all contact areas were comparable (see Fig. S12). In addition, we observed areas in the contact that seemed inaccessible to the probes (see Fig. 3 *B*). We observed spacer accumulation, implying the formation of a local gap of known height, with no QDot tracks underneath. Here, the probes were mostly deflected out of the contact, with emerging "pathways" along which probes diffuse once inside the contact. These pathways could have been indicative of membrane ruffles below the diffraction limit.

To determine whether some areas of the contact border were easier to access than others, we identified "breach zones" (i.e., parts of the boundary where QDots could successfully cross the border) (Fig. S13). Here, we found a predominantly negative correlation between entry success rate and fluorescence intensity of the spacer protein for all gap-size conditions (Fig. S13). In other words, we find lower success rates in areas of higher contact intensity, implying that the protein spacers create a physical barrier to entry simply because of the increased density of proteins in the contact versus outside. This effect of protein spacer density on the probe prompted us to study inhomogeneities of spacer protein density and the effect on probe diffusion.

QDot diffusion coefficient is reduced in a contact-density-dependent manner

Because the studied cell-bilayer contacts were not homogenous, but the distribution of the spacer protein varied across

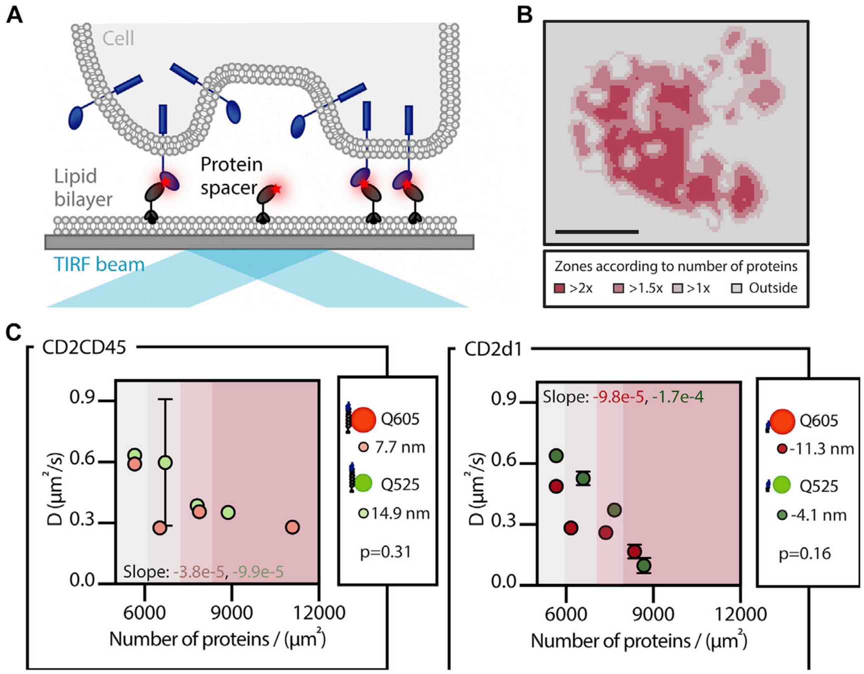

FIGURE 4 QDot diffusion influenced by spacer density. (*A*) A schematic representation of protein spacers within the contact is given. (*B*) Intensity zones of the labeled protein spacer in different areas of the contact are shown. Scale bars, 5 μm. (*C*) Diffusion coefficient (D) for QDots in areas of different protein spacer density for CD2CD45 (*left*) and CD2d1 (*right*) are shown, comparing diffusion coefficient values for constant spacer size. Slope values were analyzed using a one-way ANOVA (see Fig. S15). Values represent the mean, and error bars were obtained with boot strapping (number of iterations = 1000). If error bars are shorter than data point, error bars are not shown. Δ(P, Q) = −11.3 nm, n = 10, R^2 = 0.98, RMSE = 0.04; Δ(P, Q) = −4.1 nm, n = 6, R^2 = 0.95, RMSE = 0.06; Δ(P, Q) = 7.7 nm, n = 10, R^2 = 0.98, RMSE = 0.04; and Δ(P, Q) = 14.9 nm, n = 7, R^2 = 0.96, RMSE = 0.06.

the contact not only from cell to cell because of expression-level variation but also within each contact for a given cell, this allowed us to compare regions of different spacer protein density within one cell. To investigate whether hindered diffusion in the contact scales with spacer protein density, we studied the correlation between pixel intensity for the protein spacers and the average JD of the QDot measured for a given pixel (Fig. 4 *A*). One would expect a higher intensity to correspond with a higher density of spacer protein, implying a more stable or uniform cell-bilayer contact. In these regions, QDots should experience greater steric hindrance. To derive this correlation, we divided the contact area into regions according to their spacer protein pixel intensities and overlaid these with the JD maps (Fig. 4 *B*; see JD curves in Fig. S14). Fitting the resulting JD distributions (33), we found that the highest protein spacer density corresponded to the lowest diffusion coefficient for all gap-size conditions examined (Fig. 4, *C* and *D*). We observed an almost linear decrease in diffusion coefficient with protein density for negative Δ(P, Q). For the larger CD2CD45-mediated gap, the reduction in QDot diffusion was not as pronounced (Fig. 4 *D*). However, this effect was more pronounced when comparing the behavior of the same QDot in different gap heights. Here, the diffusion of the small QDot (Q525) was reduced by 85% when compared with QDots outside the contact for areas of negative Δ(P, Q), whereas it was only halved for regions of positive Δ(P, Q). This suggests that both the gap height and the density of adhesion proteins are involved in shaping the behavior at the contact.

Although the protein spacer density could increase steric hindrance because of crowding, it could also promote more homogenous gap height, as the spacer proteins we used were expected to be relatively rigid. Both effects (crowding and gap size) could influence diffusion and access into the contact. Because the bilayer intensities between all conditions were similar (see Fig. S1), we could compare between the conditions. Protein densities in the bilayers were measured as 5660 ± 860 proteins per μm^2 using FCS, with 10% labeled proteins in the bilayer (see Fig. S2). We found decreased diffusion for larger QDots in smaller gaps, whereas smaller probes in larger gaps seemed to be less affected by protein density (Fig. 4). This behavior most likely also applies to proteins in the T cell contact because most T cell membrane proteins are heavily glycosylated, rendering their conformation stiff and extended (41). Interestingly, Douglass and Vale identified signaling islands on T cells where important signaling proteins are reduced in their diffusion through protein-protein interactions, forming so-called microdomains (23). Here, the proteins studied did not possess an extracellular domain that could impose steric hindrances, but a slowdown of up to 50% could still be observed. In our study, we found that even without any binding effects of the probe, similar trapping effects can be observed through increased adhesion protein density. Although we studied the impact of steric effects on the extracellular domains of proteins, a similar effect of intracellular crowding due to increased protein density cannot be excluded and is of interest for future studies.

CONCLUSIONS

We present a method to study inhomogeneities in cellular contacts, demonstrated by following QDots in contacts formed by T cells interacting with an SLB at single-particle

densities. We measured the density and instantaneous velocity of QDots inside and outside contacts, determined the energy penalty to enter and exit the contact, and created spatial maps of the diffusion rates and JDs that could then be compared to the density of the protein spacers. The results provided a detailed view of the cell-bilayer contact and suggested that significant levels of trapping of large molecules could occur with relatively little exchange of molecules across the contact boundary and a significant decrease in diffusion once the contact forms. We found a gap-size-dependent energy penalty for entering contacts and a gap-size-independent penalty for exiting. Furthermore, the contact and boundary are not uniformly accessible, with accessibility scaling with spacer protein density. We also observed a protein-spacer-density-dependent slowing down of QDots across all gap sizes. This approach has the potential to reveal the likely behavior of molecules at cell contacts, elucidating how and why their diffusion and exchange with molecules outside the contact is modified and how such effects could influence signaling.

SUPPORTING MATERIAL

Supporting Material can be found online at https://doi.org/10.1016/j.bpj.2020.01.019.

AUTHOR CONTRIBUTIONS

A.H.L., K.K., and D.K. designed the experimental plan. M.A.S. provided cell lines and purified proteins. K.K. and A.H.L. performed experiments and data analysis. A.P. provided the tracking simulation code. A.H.L., K.K., J.M., A.M.S., A.P., A.W., E.J., J.H., K.F., S.F.L., S.J.D., and D.K. wrote the article.

ACKNOWLEDGMENTS

This work was supported by a Royal Society University Research Fellowship (UF120277 to S.F.L.) and a Research Professorship (RP150066 to D.K.); the EPSRC (EP/L027631/1 to A.P.); and the Wellcome Trust (098274/Z/12/Z to S.J.D.).

REFERENCES

1. Kholodenko, B. N. 2006. Cell-signalling dynamics in time and space. *Nat. Rev. Mol. Cell Biol.* 7:165–176.
2. Smith-Garvin, J. E., G. A. Koretzky, and M. S. Jordan. 2009. T cell activation. *Annu. Rev. Immunol.* 27:591–619.
3. Davis, D. M., and M. L. Dustin. 2004. What is the importance of the immunological synapse? *Trends Immunol.* 25:323–327.
4. Bromley, S. K., W. R. Burack, …, M. L. Dustin. 2001. The immunological synapse. *Annu. Rev. Immunol.* 19:375–396.
5. Springer, T. A. 1990. Adhesion receptors of the immune system. *Nature.* 346:425–434.
6. Burroughs, N. J., K. Köhler, …, D. M. Davis. 2011. Boltzmann energy-based image analysis demonstrates that extracellular domain size differences explain protein segregation at immune synapses. *PLoS Comput. Biol.* 7:e1002076.
7. Bakalar, M. H., A. M. Joffe, …, D. A. Fletcher. 2018. Size-dependent segregation controls macrophage phagocytosis of antibody-opsonized targets. *Cell.* 174:131–142.e13.
8. Felce, J. H., E. Sezgin, …, S. J. Davis. 2018. CD45 exclusion- and cross-linking-based receptor signaling together broaden FcεRI reactivity. *Sci. Signal.* 11:eaat0756.
9. Davis, S. J., and P. A. van der Merwe. 2006. The kinetic-segregation model: TCR triggering and beyond. *Nat. Immunol.* 7:803–809.
10. Davis, S. J., and P. A. van der Merwe. 1996. The structure and ligand interactions of CD2: implications for T-cell function. *Immunol. Today.* 17:177–187.
11. Sperling, A. I., J. R. Sedy, …, J. K. Burkhardt. 1998. TCR signaling induces selective exclusion of CD43 from the T cell-antigen-presenting cell contact site. *J. Immunol.* 161:6459–6462.
12. Qi, S. Y., J. T. Groves, and A. K. Chakraborty. 2001. Synaptic pattern formation during cellular recognition. *Proc. Natl. Acad. Sci. USA.* 98:6548–6553.
13. Weikl, T. R., and R. Lipowsky. 2004. Pattern formation during T-cell adhesion. *Biophys. J.* 87:3665–3678.
14. van der Merwe, P. A., P. N. McNamee, …, S. J. Davis. 1995. Topology of the CD2-CD48 cell-adhesion molecule complex: implications for antigen recognition by T cells. *Curr. Biol.* 5:74–84.
15. Leupin, O., R. Zaru, …, S. Valitutti. 2000. Exclusion of CD45 from the T-cell receptor signaling area in antigen-stimulated T lymphocytes. *Curr. Biol.* 10:277–280.
16. Cai, H., J. Muller, …, S. J. Wind. 2018. Full control of ligand positioning reveals spatial thresholds for T cell receptor triggering. *Nat. Nanotechnol.* 13:610–617.
17. Irvine, D. J., M. A. Purbhoo, …, M. M. Davis. 2002. Direct observation of ligand recognition by T cells. *Nature.* 419:845–849.
18. Huang, J., M. Brameshuber, …, M. M. Davis. 2013. A single peptide-major histocompatibility complex ligand triggers digital cytokine secretion in CD4(+) T cells. *Immunity.* 39:846–857.
19. Limozin, L., M. Bridge, …, P. Robert. 2019. TCR-pMHC kinetics under force in a cell-free system show no intrinsic catch bond, but a minimal encounter duration before binding. *Proc. Natl. Acad. Sci. USA.* 116:16943–16948.
20. Lin, J. J. Y., S. T. Low-Nam, …, J. T. Groves. 2019. Mapping the stochastic sequence of individual ligand-receptor binding events to cellular activation: T cells act on the rare events. *Sci. Signal.* 12:eaat8715.
21. Huppa, J. B., M. Axmann, …, M. M. Davis. 2010. TCR-peptide-MHC interactions in situ show accelerated kinetics and increased affinity. *Nature.* 463:963–967.
22. Sibener, L. V., R. A. Fernandes, …, K. C. Garcia. 2018. Isolation of a structural mechanism for uncoupling T cell receptor signaling from peptide-MHC binding. *Cell.* 174:672–687.e27.
23. Douglass, A. D., and R. D. Vale. 2005. Single-molecule microscopy reveals plasma membrane microdomains created by protein-protein networks that exclude or trap signaling molecules in T cells. *Cell.* 121:937–950.
24. Dietrich, C., B. Yang, …, K. Jacobson. 2002. Relationship of lipid rafts to transient confinement zones detected by single particle tracking. *Biophys. J.* 82:274–284.
25. Guo, Y., D. Li, …, D. Li. 2018. Visualizing intracellular organelle and cytoskeletal interactions at nanoscale resolution on millisecond timescales. *Cell.* 175:1430–1442.e17.
26. Dustin, M. L., M. W. Olszowy, …, A. S. Shaw. 1998. A novel adaptor protein orchestrates receptor patterning and cytoskeletal polarity in T-cell contacts. *Cell.* 94:667–677.
27. Schmid, E. M., M. H. Bakalar, …, D. A. Fletcher. 2016. Size-dependent protein segregation at membrane interfaces. *Nat. Phys.* 12:704–711.
28. Cartwright, A. N. R., J. Griggs, and D. M. Davis. 2014. The immune synapse clears and excludes molecules above a size threshold. *Nat. Commun.* 5:5479.

29. Alakoskela, J. M., A. L. Koner, …, D. M. Davis. 2011. Mechanisms for size-dependent protein segregation at immune synapses assessed with molecular rulers. *Biophys. J.* 100:2865–2874.

30. Cai, E., K. Marchuk, …, M. F. Krummel. 2017. Visualizing dynamic microvillar search and stabilization during ligand detection by T cells. *Science.* 356:eaal3118.

31. Beemiller, P., J. Jacobelli, and M. F. Krummel. 2012. Integration of the movement of signaling microclusters with cellular motility in immunological synapses. *Nat. Immunol.* 13:787–795.

32. Waithe, D., M. P. Clausen, E. Sezgin, and C. Eggeling. 2016. FoCuS-point: software for STED fluorescence correlation and time-gated single photon counting. *Bioinformatics.* 32:958–960.

33. Weimann, L., K. A. Ganzinger, …, D. Klenerman. 2013. A quantitative comparison of single-dye tracking analysis tools using Monte Carlo simulations. *PLoS One.* 8:e64287.

34. Sonnleitner, A., G. J. Schütz, and T. Schmidt. 1999. Free Brownian motion of individual lipid molecules in biomembranes. *Biophys. J.* 77:2638–2642.

35. Choudhuri, K., D. Wiseman, …, P. A. van der Merwe. 2005. T-cell receptor triggering is critically dependent on the dimensions of its peptide-MHC ligand. *Nature.* 436:578–582.

36. James, J. R., and R. D. Vale. 2012. Biophysical mechanism of T-cell receptor triggering in a reconstituted system. *Nature.* 487:64–69.

37. Chang, V. T., R. A. Fernandes, …, S. J. Davis. 2016. Initiation of T cell signaling by CD45 segregation at 'close contacts'. *Nat. Immunol.* 17:574–582.

38. Marino, M., D. I. Svergun, …, O. Mayans. 2005. Poly-Ig tandems from I-band titin share extended domain arrangements irrespective of the distinct features of their modular constituents. *J. Muscle Res. Cell Motil.* 26:355–365.

39. Carbone, C. B., N. Kern, …, R. D. Vale. 2017. In vitro reconstitution of T cell receptor-mediated segregation of the CD45 phosphatase. *Proc. Natl. Acad. Sci. USA.* 114:E9338–E9345.

40. Przybylo, M., J. Sýkora, …, M. Hof. 2006. Lipid diffusion in giant unilamellar vesicles is more than 2 times faster than in supported phospholipid bilayers under identical conditions. *Langmuir.* 22:9096–9099.

41. Jentoft, N. 1990. Why are proteins O-glycosylated? *Trends Biochem. Sci.* 15:291–294.

Biophysical Journal, Volume *118*

Supplemental Information

The Costs of Close Contacts: Visualizing the Energy Landscape of Cell Contacts at the Nanoscale

Klara Kulenkampff, Anna H. Lippert, James McColl, Ana Mafalda Santos, Aleks Ponjavic, Edward Jenkins, Jane Humphrey, Alexander Winkel, Kristian Franze, Steven F. Lee, Simon J. Davis, and David Klenerman

Supporting Material

The costs of close contacts: Visualizing the energy landscape of cell contacts at the nanoscale

K. Kulenkampff [1]*, A. H. Lippert[1]*, J. McColl[1], M. A. Santos[2], A. Ponjavic[1], Edward Jenkins[2], Jane Humphrey[1], Alexander Winkel[3], Kristian Franze[3], S. F. Lee[1], S. J. Davis[2], D. Klenerman[1]

[1] Department of Chemistry, University of Cambridge, Cambridge, United Kingdom
[2] Radcliffe Department of Medicine and MRC Human Immunology Unit, John Radcliffe Hospital, University of Oxford, Oxford, United Kingdom
[3] Department of Physiology, Development and Neuroscience, University of Cambridge, Cambridge, United Kingdom

Suppl. Video 1: Measuring unspecific binding of QDots to cells in HILO mode

Suppl. Video 2A and 2B: QDots 605 diffusing in a bilayer and through a CD2rCD45 cell contact. (A) Raw video of QDots diffusing in bilayer. (B) Time-lapse of tracks colour-coded according to track speed (analysed with TrackMate by Fiji)

Suppl. Video 3: QDots 525 diffusing in a bilayer and through a CD2rCD45 cell contact

Suppl. Video 4A and 4B: Simulation of QDots diffusing freely in a bilayer and through a CD45 cell contact. (A) Tracks starting inside the cell contact. (B) Tracks starting outside the cell contact

Suppl. Table 1: Number of tracks attempting to enter or exit the cell contact and success to enter or exit.

Suppl. Fig. 1: Variation of bilayer intensity for different conditions

Suppl. Fig. 2: FCS measurements of protein densities

Suppl. Fig. 3: Unspecific binding of QDots to cells is minor.

Suppl. Fig. 4: Average tracklength in frames for each cell measured

Suppl. Fig. 5: Average signal-to-noise-ratio for each cell measured

Suppl. Fig. 6: QDots diffuse freely in bilayer (MSD plot)

Suppl. Fig. 7: Contacts are mediated by rat CD2 and rat CD48 constructs.

Suppl. Fig. 8: Shift of JD distribution between attempts, exit and enter

Suppl. Fig. 9: Energy penalties for eroded and dilated contact borders

Suppl. Fig. 10: Maps of number of counts and jump distance per pixel

Suppl. Fig. 11: ECDFs of overall jump distances in track image inside the contacts

Suppl. Fig. 12: Contact sizes between gap conditions similar

Suppl. Fig. 13: Negative correlation between success rate and spacer pixel intensity

Suppl. Fig. 14: JD distributions separated out via spacer intensity zones

Suppl. Fig. 15: Additional statistics for linear fit of diffusion constants in areas of various protein density

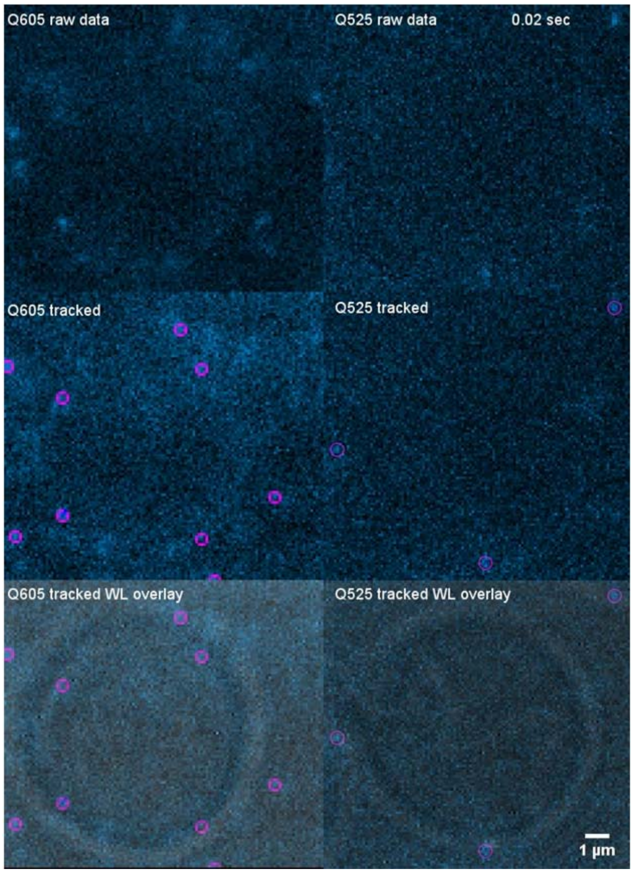

Supplementary Video 1: Measuring unspecific binding of QDots to cells in HILO mode. Detected QDots are marked with a purple circle.

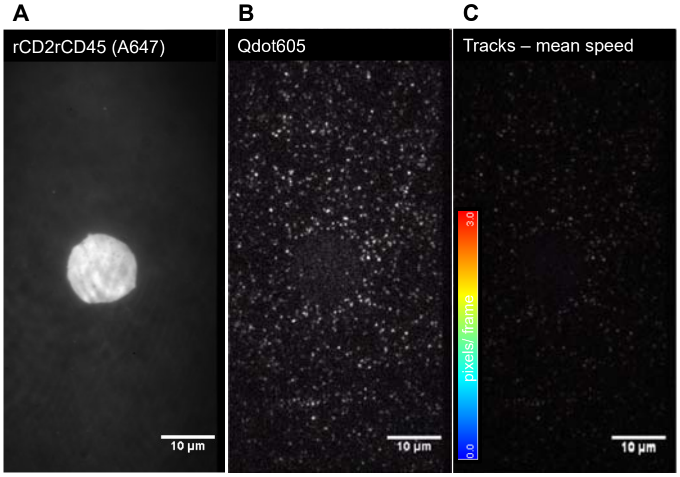

Supplementary Video 2: Raw data and tracks of Q605s attached to a SLB. Cell is immobilised by the protein spacer rCD2rCD45. (A) Average image over 500 frames of the labelled protein spacer. (B) Raw time-lapse data of the channel showing the QDots. (C) Time-lapse of tracks colour-coded according to track speed (analysed with TrackMate by Fiji).

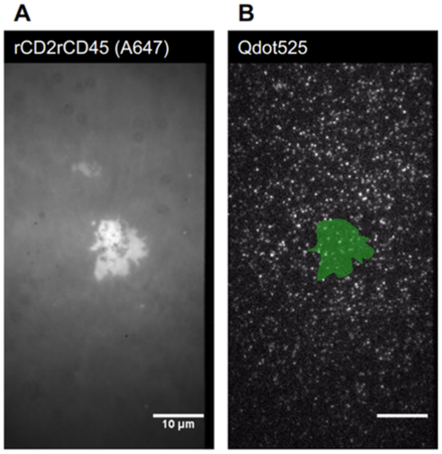

Supplementary Video 3: Raw data of Q525s attached to a SLB. Cell is again immobilised by the protein spacer rCD2rCD45. (A) Average image over 500 frames of the labelled protein spacer. (B) Raw time-lapse data of the channel showing the QDots. The green mask indicates the approximate position of the cell.

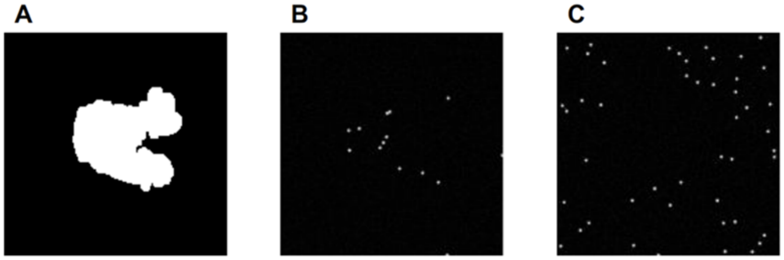

Supplementary Video 4: Simulation of QDots diffusing through a cell contact. (A) One of the cell boarders used to create simulation data. (B) Simulation where QDots start indside the cell contact and freely diffuse outof and within the contact. (C) Simulation where QDots start outside the cell contact and freely diffuse into and outside the contact zone.

	Standard				Dilate				Erode				
Δ(P,Q) (nm)	-11.3	-4.1	7.7	14.9	-11.3	-4.1	7.7	14.9	-11.3	-4.1	7.7	14.9	Sim
Number of attempts to enter	197	191	452	240	155	204	410	247	13	19	92	48	650
Number of attempts to exit	27	44	160	118	159	177	433	322	8	13	62	36	225

Supplementary Table 1: Number of tracks attempting to enter or exit the cell contact and success to enter or exit.

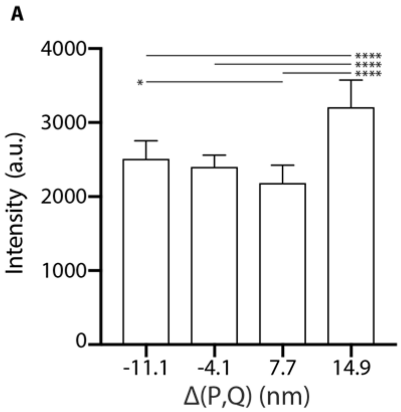

B

Intensity zone	Δ(P,Q) (nm)							
	-11.1		-4.1		7.7		14.9	
	Mean	Std	Mean	Std	Mean	Std	Mean	Std
Outside	2711	283	2981	184	3117	289	3988	418
(1-1.5)	3235	185	3455	197	3558	218	4665	929
(1.5-2)	3862	199	4022	182	4300	214	5428	796
> 2	4383	159	4555	40	6050	740	6168	601

Supplementary Figure 1: (A) Mean intensities of protein spacers outside the cell contact. P values were obtained from a one-way ANOVA test. (B) Mean intensities of different intensity zones inside and outside the cell contact. The intensity zone (1-1.5, 1.5-2.0 and more than 2.0 times the outside intensity) are within the cell contact.

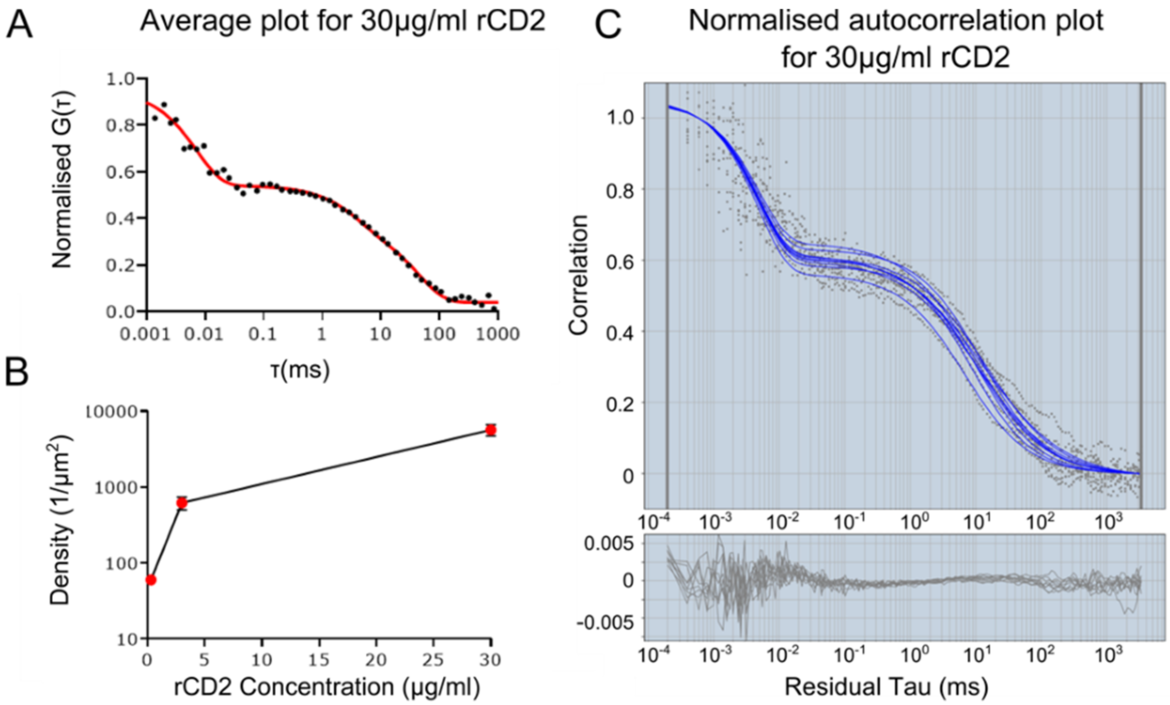

Supplementary Figure 2: Protein density determination using FCS. A) Average plot for 30 µg/ml rCD2. Measurements were performed using underlabelled conditions since FCS curves could not be obtained at a concentration of 30 µg/ml because the sensor was saturated preventing any accurate readings. Instead we used a mixture of labelled and unlabelled rCD2 (10% labelled Alexa 647 and 90% unlabelled). The obtained density was then multiplied by 10 to give the 'true' density reading. Three FCS readings (10 s each) were taken on three bilayers for each concentration of rCD2 tested (9 FCS readings total per concentration). FCS data was analysed using FoCuS-point (https://www.ncbi.nlm.nih.gov/pubmed/26589275). B) Protein density measured at different rCD2 concentrations measured via FCS. C) Normalised autocorrelation for 30 µg/ml rCD2.

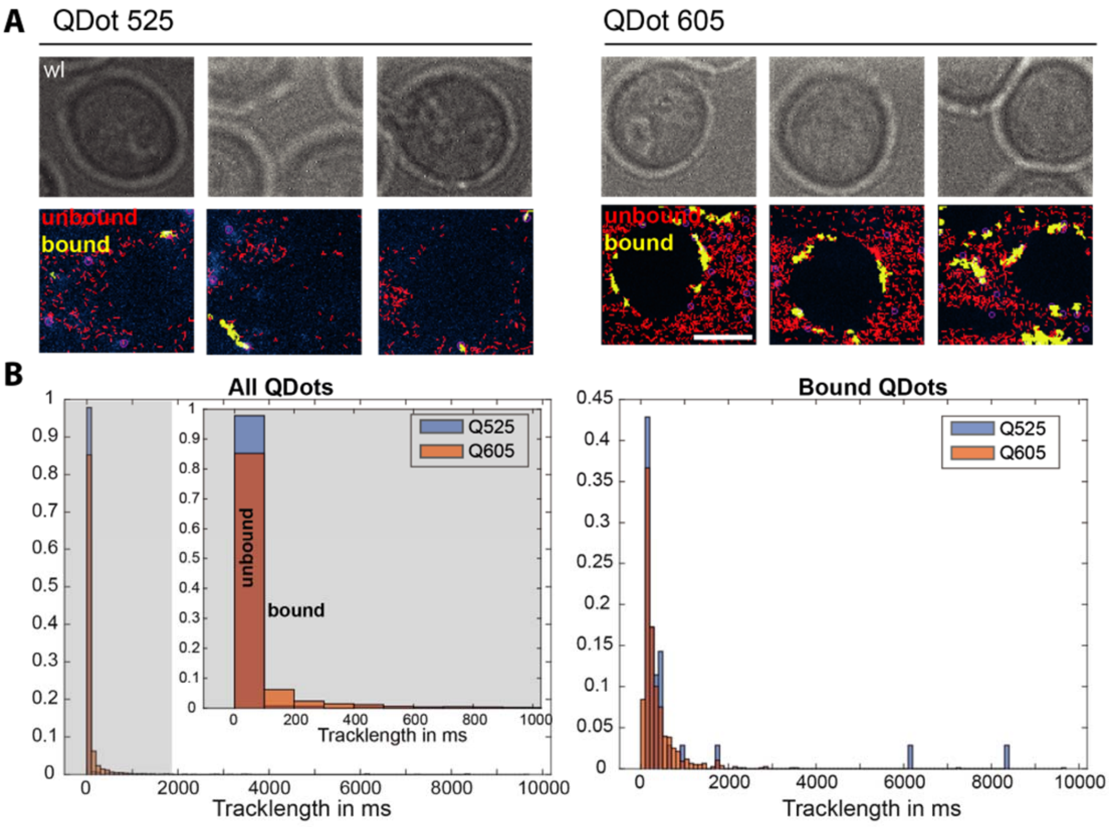

C

	Q525	Q605
**tracks selected as bound** (tracks longer than 5 frames at 20ms frame rate = 100ms)	4.7% ±3.7%	11.4% ±5.1%
**tracks relevant for analysis** (tracks longer than 5 frames at 60ms frame rate = 300ms)	1.8% ±2.1%	4.9% ±2.2%

Supplementary Figure 3: Unspecific binding of QDots to cells is minor. Jurkat rCD48 cells were incubated with QDots at a concentration of 500 nM. Cells were imaged in HILO to observe unspecific, temporary binding of QDots to cells at high frame rates. (A) The upper panel shows the white light and QDot channel with tracks coloured in bound (yellow) vs unbound (red). Scale bar, 5 µm. (B) Histograms of tracklengths of all QDots trackable in the video (All QDots, left). Tracks longer than five frames were classified as bound (right). Data was collected from five cells per condition. (C) Table gives mean ± SD percentage of QDots bound longer than five frames as indication of temporary non-specific interactions (tracks selected as bound). Since analysis rejects tracks shorter than five frames the only relevant tracks are those bound for longer than 300 ms (tracks relevant for analysis).

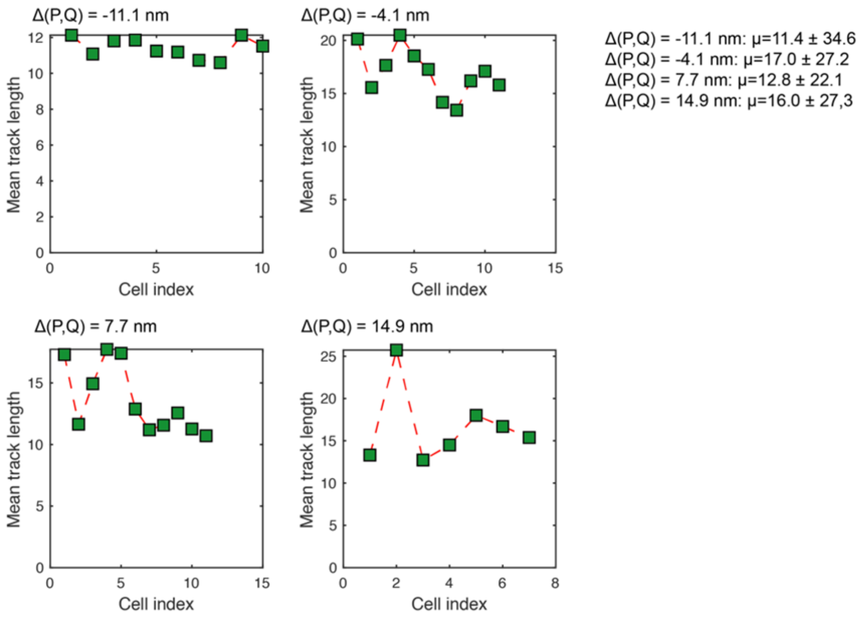

Supplementary Figure 4: Average tracklength in frames for each cell measured. μ states the mean tracklength per condition.

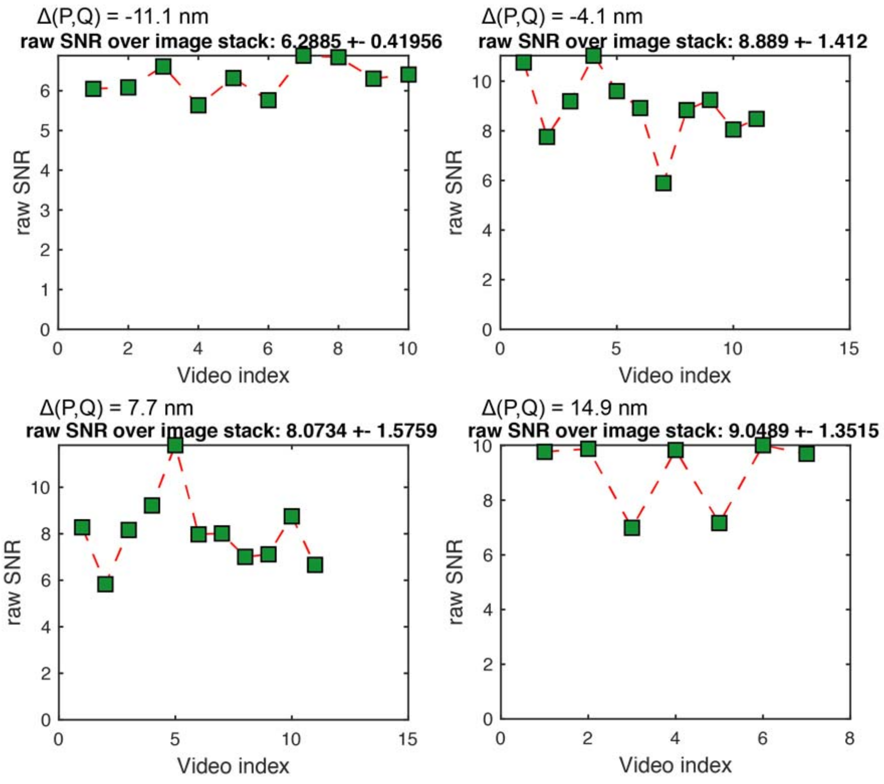

Supplementary Figure 5: Average signal-to-noise-ratio for each cell measured.

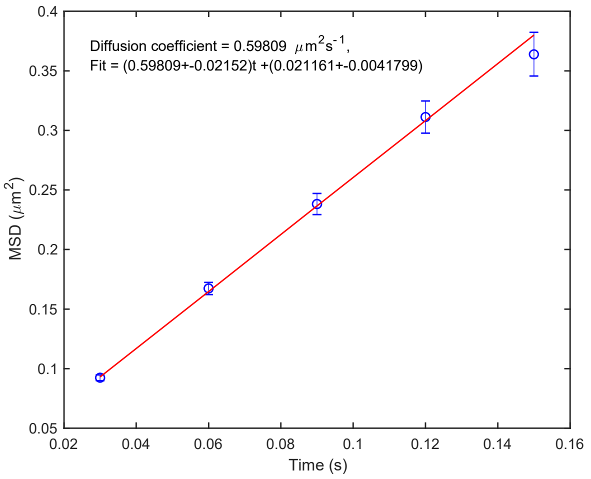

Supplementary Figure 6: MSD plot of QDots (Q605) freely diffusing in bilayers. The MSD curve was fitted using a custom-written Matlab code by Weimann *et al.*(33)

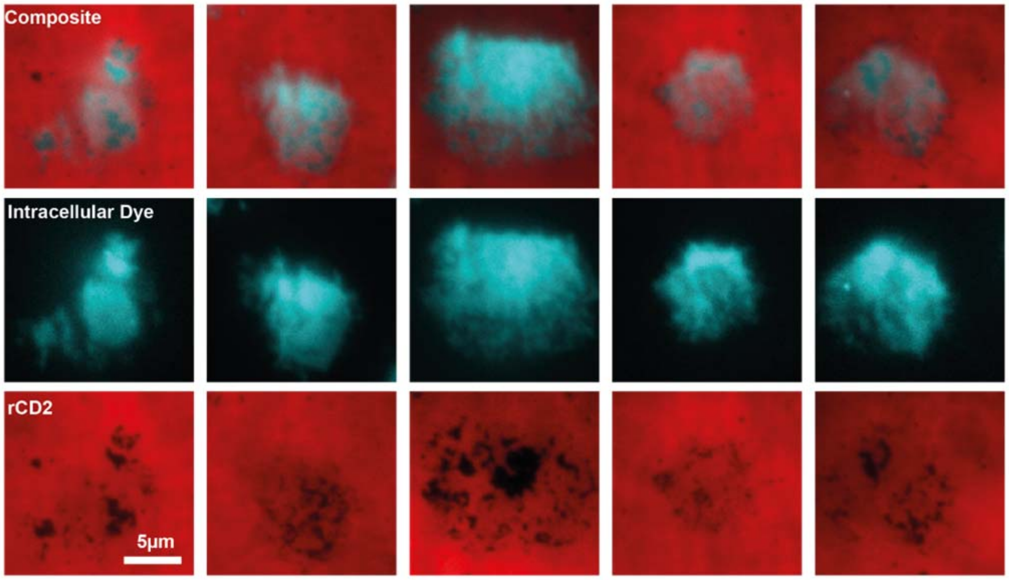

Supplementary Figure 7: Contacts are mediated by ratCD2 and rat CD48 constructs. Expression of rCD48 is necessary for rCD2 accumulation. Jurkat cells which do not express rCD48 were dropped on rCD2 (Alexa647) bilayers. To indicate the position of the cells without leading to membrane dye leakage into the bilayer, cells were labelled intracellularly with Fluo4 dye.

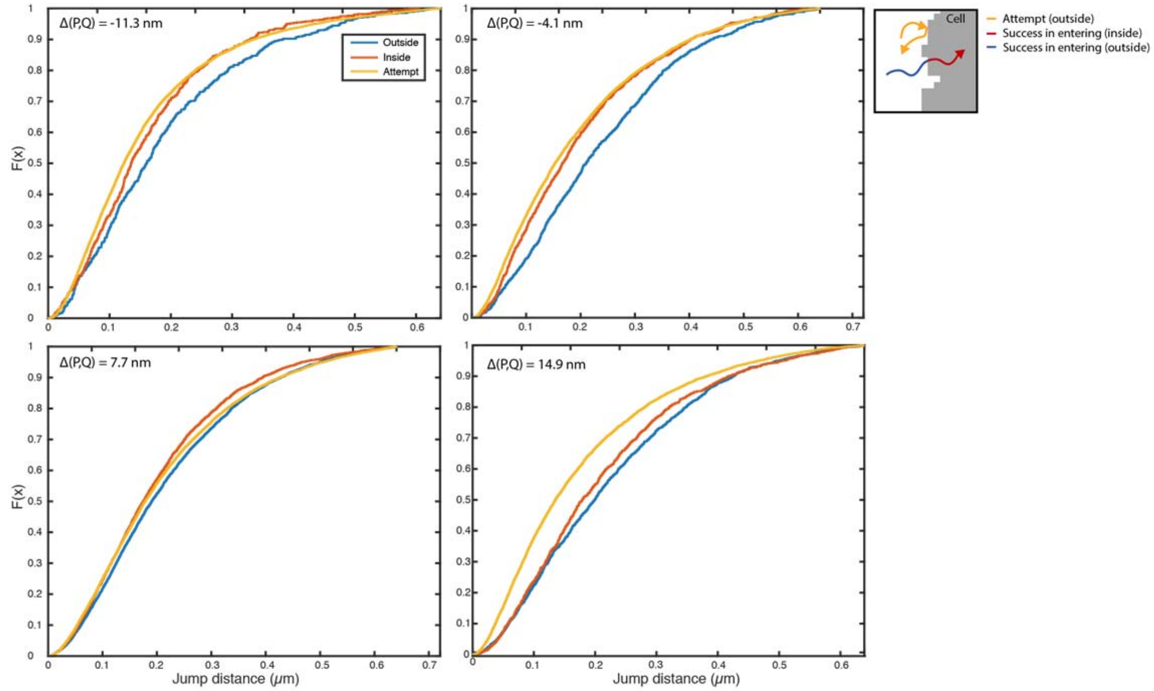

Supplementary Figure 8: JD distributions for different gap conditions for successful tracks, separated out according to being inside or outside the contact. Blue curves correspond to JD values from the outside part of QDot tracks which successfully entered the contact, red curves are the JD values of the tracks inside the contact, after successfully entering the contact. The yellow curves are JD values from tracks attempting but failing to enter the contact.

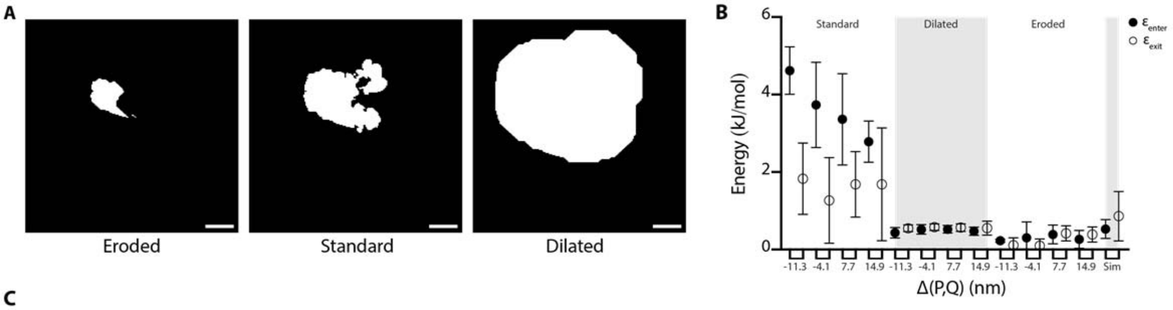

C

		Standard entering				Standard exiting				Dilate entering				Dilate exiting				Erode entering				Erode exiting					
	Δ(P,Q) (nm)	-11.3	-4.1	7.7	14.9	-11.3	-4.1	7.7	14.9	-11.3	-4.1	7.7	14.9	-11.3	-4.1	7.7	14.9	-11.3	-4.1	7.7	14.9	-11.3	-4.1	7.7	14.9	Sim entering	Sim exiting
Standard enteri	-11.3		ns	**	****	****	****	****	****	****	****	****	****	****	****	****	****	****	****	****	****	****	****	****	****	****	****
	-4.1	ns		ns	ns	***	****	****	****	****	****	****	****	****	****	****	****	****	****	****	****	****	****	****	****	****	****
	7.7	**	ns		ns	***	****	****	****	****	****	****	****	****	****	****	****	****	****	****	****	****	****	****	****	****	****
	14.9	****	ns	ns		ns	**	*	ns	****	****	****	****	****	****	****	****	****	****	****	****	****	****	****	****	****	****
Standard exiting	-11.3	****	***	***	ns		ns	ns	ns	**	ns	*	*	*	ns	*	*	**	*	**	**	*	*	**	**	**	ns
	-4.1	****	****	****	**	ns		ns	ns	ns	ns	ns	ns	ns	ns	ns	ns	ns	ns	ns	ns	ns	ns	ns	ns	ns	ns
	7.7	****	****	****	*	ns	ns		ns	***	*	**	**	**	ns	**	*	ns	**	***	***	*	*	**	**	***	ns
	14.9	****	****	****	ns	ns	ns	ns		**	ns	*	*	*	ns	*	ns	ns	*	**	**	*	*	**	*	**	ns
Dilate entering	-11.3	****	****	****	****	**	ns	***	**		ns	ns	ns	ns	ns	ns	ns	ns	ns	ns	ns	ns	ns	ns	ns	ns	ns
	-4.1	****	****	****	****	ns	ns	*	ns	ns		ns	ns	ns	ns	ns	ns	ns	ns	ns	ns	ns	ns	ns	ns	ns	ns
	7.7	****	****	****	****	*	ns	**	*	ns	ns		ns	ns	ns	ns	ns	ns	ns	ns	ns	ns	ns	ns	ns	ns	ns
	14.9	****	****	****	****	*	ns	**	*	ns	ns	ns		ns	ns	ns	ns	ns	ns	ns	ns	ns	ns	ns	ns	ns	ns
Dilate exiting	-11.3	****	****	****	****	*	ns	**	*	ns	ns	ns	ns		ns	ns	ns	ns	ns	ns	ns	ns	ns	ns	ns	ns	ns
	-4.1	****	****	****	****	ns	ns	ns	ns	ns	ns	ns	ns	ns		ns	ns	ns	ns	ns	ns	ns	ns	ns	ns	ns	ns
	7.7	****	****	****	****	*	ns	**	*	ns	ns	ns	ns	ns	ns		ns	ns	ns	ns	ns	ns	ns	ns	ns	ns	ns
	14.9	****	****	****	****	*	ns	*	ns	ns	ns	ns	ns	ns	ns	ns		ns	ns	ns	ns	ns	ns	ns	ns	ns	ns
Erode entering	-11.3	****	****	****	****	**	ns	ns	ns	ns	ns	ns	ns	ns	ns	ns	ns		ns	ns	ns	ns	ns	ns	ns	ns	ns
	-4.1	****	****	****	****	*	ns	**	*	ns	ns	ns	ns	ns	ns	ns	ns			ns	ns	ns	ns	ns	ns	ns	ns
	7.7	****	****	****	****	**	ns	***	**	ns	ns	ns	ns	ns	ns	ns	ns	ns	ns		ns	ns	ns	ns	ns	ns	ns
	14.9	****	****	****	****	**	ns	***	**	ns	ns	ns	ns	ns	ns	ns	ns	ns	ns	ns		ns	ns	ns	ns	ns	ns
Erode exiting	-11.3	****	****	****	****	*	ns	*	*	ns	ns	ns	ns	ns	ns	ns	ns	ns	ns	ns	ns		ns	ns	ns	ns	ns
	-4.1	****	****	****	****	*	ns	*	*	ns	ns	ns	ns	ns	ns	ns	ns	ns	ns	ns	ns	ns		ns	ns	ns	ns
	7.7	****	****	****	****	**	ns	**	**	ns	ns	ns	ns	ns	ns	ns	ns	ns	ns	ns	ns	ns	ns		ns	ns	ns
	14.9	****	****	****	****	**	ns	**	*	ns	ns	ns	ns	ns	ns	ns	ns	ns	ns	ns	ns	ns	ns	ns		ns	ns
	Sim entering	****	****	****	****	**	ns	***	**	ns	ns	ns	ns	ns	ns	ns	ns	ns	ns	ns	ns	ns	ns	ns	ns		ns
	Sim exiting	****	****	****	****	ns	ns	ns	ns	ns	ns	ns	ns	ns	ns	ns	ns	ns	ns	ns	ns	ns	ns	ns	ns	ns	

Supplementary Figure 9: Energy penalties for experimental, eroded and dilated contact borders. (A) Contact masks used to test success rate of QDots entering and exiting an 'artificial' contact, scale bars 5 µm. (B) Energy penalties for entering and exiting eroded, real and dilated contacts. C) Table of p-Values comparing all gap conditions with all eroded, dilated, simulated and experimental contacts.

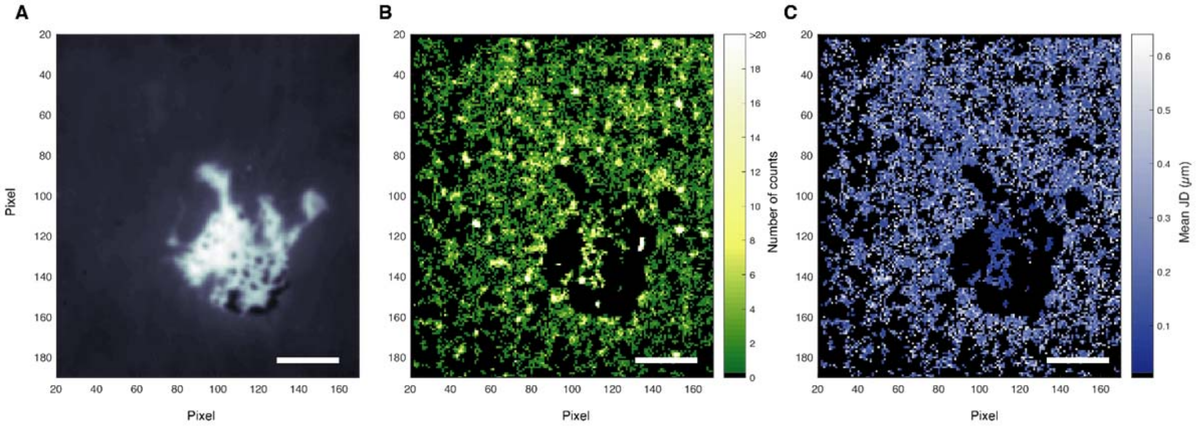

Supplementary Figure 10: (A) Fluorescence image of a cell binding to CD2CD45 in a SLB. Scale bar, 5 µm. (B) Map showing the number of counts per pixel of a CD2CD45 / QD605 contact. Mean number of counts per pixel µ=5.2 ±11.9. (C) Corresponding map of jump distances.

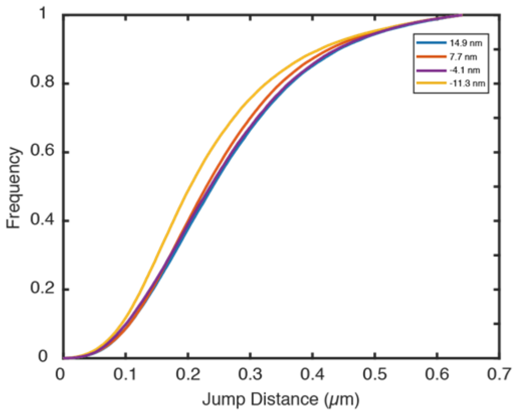

Supplementary Figure 11: ECDFs of overall jump distances in track image inside the contacts.

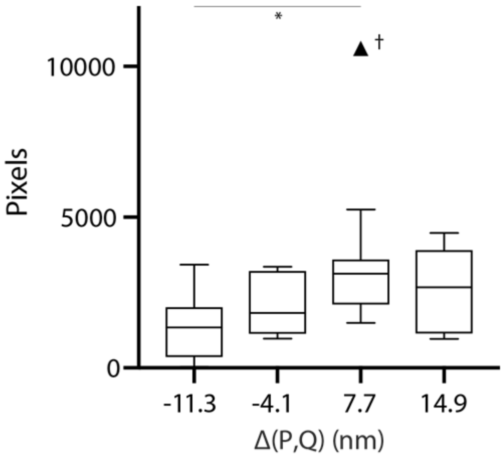

Supplementary Figure 12: Contact size distribution in pixel for each condition. A one-way Anova comparing the means of each condition did not show a significant difference, other than between Δ(P,Q) = -11.3 nm and 7.7 nm. †This data point shows a field of view containing two cells.

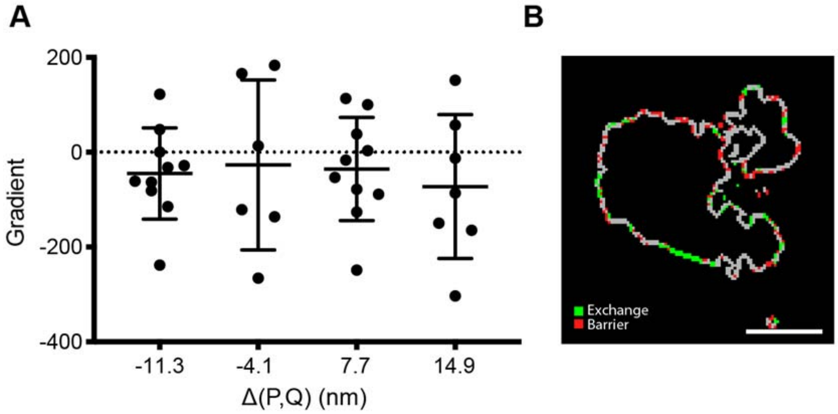

Supplementary Figure 13: Gradient coefficient for each linear fit for the success rate plotted against the intensity at that pixel for all border pixels. The success rate was weighted to the number of attempts to enter at the position of the pixel. B) Representative border image of a rCD2rCD45 Contact probed with a QDot605 spacer. Pixels in the border where no exchange occurred are marked in white, pixels where probes where reflected off are marked red and pixels where probes exchanged over the barriers are marked green. Scale bar 5 µm.

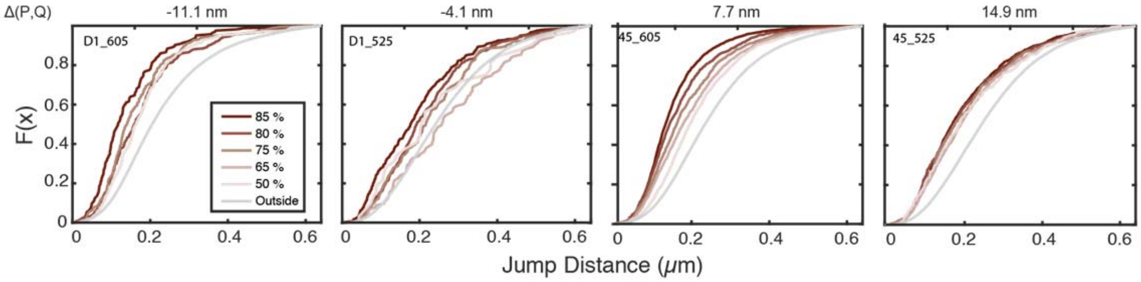

Supplementary Fig 14: ECDFs of JD populations separated via intensity zones for different gap conditions

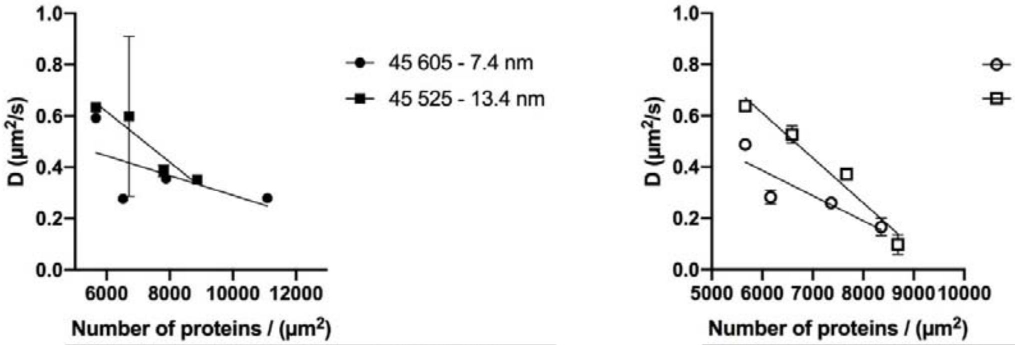

	45 605, 7.7 nm	45 525, 14.9 nm
Slope	-3.836e-005	-9.856e-005
Y-intercept	0.6747	1.208

	D1 605, -11.1 nm	D1 525, -5.1 nm
Slope	-9.820e-005	-0.0001754
Y-intercept	0.9759	1.663

Supplementary Figure 15: Results for linear fit of diffusion constants in areas of protein number.
